# Developing drugs targeting CX3CL1 to treat heart diseases via immune/inflammatory mediation: Targeting CX3CL1 to treat heart diseases

**DOI:** 10.3724/abbs.2023157

**Published:** 2023-08-14

**Authors:** Lin Zou, Junhua Ma, Guiying Hu, Hongling Zhu, Lijuan Zhang, Xiangqi Li

**Affiliations:** 1 Department of Endocrinology and Metabolism Gongli Hospital Naval Medical University Shanghai 200135 China; 2 Outpatient Department Zhongxian People’s Hospital Chongqing 404300 China; 3 Department of Endocrinology Shanghai Songjiang District Center Hospital Shanghai 201600 China

Heart dysfunction can be induced by nonheart or external stresses, including environmental exposures, socioeconomic position [1], physical inactivity [2], and numerous nonheart diseases, such as cancers, diabetes, chronic kidney disease, metabolic syndromes, sepsis, thyroid disease, and aging. In particular, COVID-19 stress yields cardiac abnormalities, often leading to mortality [3]. Undoubtedly, these diseases and environmental exposures all share the same biological factor, inflammation/immunity. Indeed, an intriguing novel concept shows that inflammation is an intermediate biological risk factor for heart disease [4]. The heart is referred to as an immune organ due to its response to tissue injury through complex inflammatory events [5]. As such, inflammatory/immune disorders may be a bridge between the cardiac response and external stresses. CX3CL1/Fractalkine belongs to the CX3C subgroup of chemokines and plays roles in almost all common diseases. To date, approximately 2000 papers (PubMed) have been published focusing on targeting CX3CL1 to investigate disease development, most of which are associated with inflammatory/immune disorders. We propose that CX3CL1 may be a crucial factor bridging the heart response and nonheart stress by its involvement in the inflammatory/immune pathway.

To support this hypothesis, we used big data from many excellent online databases and tools to mine the CX3CL1-centred molecular network. Expression analysis, coexpression dissection, ceRNA (competing endogenous RNA) extraction, intersection gene decoding, functional commenting, and chemical targeting were performed. For all analyses, unless otherwise stated, we used the default parameters. Figures were prepared using Excell, PPT, online tools, or drawing software. Detailed methods are provided in Supplementary Data 1, and the analyzed molecules can be found in Supplementary Tables S1–3.

We first revealed the inflammation/immunity-related roles of CX3CL1 in the heart by gene coexpression analysis employing GEPIA2 and DAVID. For GO-BP, we obtained 132 terms and presented the top 30 terms ( Supplementary Figure S1A). Most of them focus on inflammation/immunity-related terms. GO-CC has 18 terms, showing widely distributed expression in the cell or extracellular space ( Supplementary Figure S1B). CX3CL1 is also found in the NF-κB(2) complex, an inflammation/immunity-related complex. For GO-MF, 29 terms were compiled, and obvious inflammation/immunity-related functions, such as “virus receptor activity” and “MHC class Ib protein binding”, were found ( Supplementary Figure S1C). For KEGG, 35 terms were obtained, and many of them are virus infection-related pathways ( Supplementary Figure S1D), which was further confirmed by HIV interaction analysis ( Supplementary Figure S1E). Next, tissue expression analysis showed wide expression in human tissues and immunity-related cells such as “monocytes”, “lymph”, and “leukocytes” ( Supplementary Figure S1F). Furthermore, GAD DISEASE analysis demonstrated the involvement of CX3CL1 in “immune”, “infection”, and “cardiovascular” diseases ( Supplementary Figure S1G).

Then, we found extensive functions of CX3CL1 in tissues outside the heart by ceRNA analysis. Using ENCORI, we identified 1782 genes sharing ceRNA mechanisms with CX3CL1 in 32 human tissues (except the heart) to carry out functional commenting using DAVID. We obtained 277 functional terms of GO-BP and found that CX3CL1 may be involved in various biological processes, including “viral process” ( Supplementary Figure S2A). GO-CC analysis revealed 94 terms, showing its wide expression in various suborganelles ( Supplementary Figure S2B). For GO-MF, we identified 96 terms and found various molecular functions of CXC3CL1 ( Supplementary Figure S2C). By KEGG commenting, we acquired 83 terms, including many infection-related annotations, such as “Hepatitis C”, “HTLV-I infection”, “Shigellosis”, and “Hepatitis B” ( Supplementary Figure S2D). Further analysis of HIV infection showed the involvement of CX3CL1 in infection events ( Supplementary Figure S2E). Extensive functions of CX3CL1 were further verified by GAD DISEASE analysis ( Supplementary Figure S2F), in which 57 functional disease annotations were obtained, including heart diseases such as “coronary artery disease” and “sudden cardiac death” as well as inflammation-related diseases such as “chronic obstructive pulmonary disease”. Thirty-nine terms from tissue expression analysis also supported its extensive functions ( Supplementary Figure S2G).

Next, we posed functions of intersection genes between ceRNA and coexpression genes of CX3CL1. ceRNA genes and coexpression genes of CX3CL1 were enlisted to mine intersection genes, and 23 cross genes were obtained ( Supplementary Figure S3A). Furthermore, we implemented Gene Ontology (GO), KEGG, HIV interaction and tissue expression analyses ( Supplementary Figure S3B) and obtained 6 genes, including *CCL8*, *IL15RA*, *MAP3K11*, *APOL3*, *CSF1*, and *ICOSLG*, which are obviously associated with immunity/inflammation-related terms. Such roles were also confirmed by their annotations in the immune database ( Supplementary Figure S3C). Because the GO terms in the NCBI online repository are from published reports, we queried these 6 genes one by one in NCBI ( Supplementary Figure S3D–I) and obtained many functional annotations. Some of these notes confirmed their immunity/inflammation-related functions. For example, CCL8 has functional terms such as “inflammatory response”, “immune response”, and “response to virus” ( Supplementary Figure S3D); ICOSLG has “defense response”, “T-cell costimulation”, “positive regulation of activated T-cell proliferation”, “T-cell activation”, and “B-cell activation” ( Supplementary Figure S3E). CSF1 has “cytokine activity”, “macrophage colony-stimulating factor receptor binding”, “inflammatory response”, “macrophage differentiation”, “monocyte activation”, “innate immune response”, “positive regulation of macrophage differentiation”, and “positive regulation of monocyte differentiation” ( Supplementary Figure S3G); IL15RA has “cytokine receptor activity”, “cytokine-mediated signaling pathway”, and “positive regulation of natural killer cell differentiation” ( Supplementary Figure S3H); and APOL3 has “inflammatory response” and “positive regulation of I-kappaB kinase/NF-kappaB signaling” ( Supplementary Figure S3I).

We further extracted critical molecules to form a ceRNA network by addressing the expression atlas and functional effects of intersecting genes and miRNA genes for CX3CL1. As mentioned above, 6 intersecting genes form ceRNAs with CX3CL1. Thus, we want to know which miRNA(s) is/are the bridge between CX3CL1 and these 6 intersection genes. CCL8 binds to 10 miRNAs, CSF1 to 78 miRNAs, ICOSLG to 74 miRNAs, IL15RA to 26 miRNAs, MAP3K11 to 55 miRNAs, and APOL3 to 14 miRNAs ( Supplementary Figure S4A). Eleven of these miRNAs share up to 4 genes among the six genes, and such miRNAs are hsa-miR-150-5p, hsa-miR-214-3p, hsa-miR-27a-3p, hsa-miR-27b-3p, hsa-miR-296-5p, hsa-miR-342-3p, hsa-miR-3619-5p, hsa-miR-4731-5p, hsa-miR-513a-5p, hsa-miR-532-3p, and hsa-miR-761 ( Supplementary Figure S4B). Next, we retrieved the expression atlas of these miRNAs in all parts of the heart. hsa-miR-27a-3p and hsa-miR-27b-3p have high expression; hsa-miR-150-5p, hsa-miR-214-3p, and hsa-miR-342-3p present medium expression; and hsa-miR-296-5p, hsa-miR-532-3p, hsa-miR-3619-5p, hsa-miR-513a-5p, hsa-miR-4731-5p, and hsa-miR-761 show a low level or no expression ( Supplementary Figure S5). Furthermore, KEGG pathways were dissected for these miRNAs, and 9 of them have annotated information. Hsa-miR-150-5p is enriched in virus infection terms in addition to cancers and in “arrhythmogenic right ventricular cardiomyopathy (ARVC)” ( Supplementary Figure S6A). Hsa-miR-214-3p is involved in myeloid leukemia and arrhythmogenic right ventricular cardiomyopathy (ARVC) ( Supplementary Figure S6B). Hsa-miR-27a-3p participates in “viral carcinogenesis”, “bacterial invasion of epithelial cells”, “chronic myeloid leukemia”, “shigellosis”, and “hepatitis B” ( Supplementary Figure S6C). Hsa-miR-27b-3p also participates in “viral carcinogenesis”, “bacterial invasion of epithelial cells”, and “chronic myeloid leukemia” ( Supplementary Figure S6D). Another 5 miRNAs have no obvious infection- or immunity-related terms ( Supplementary Figure S6E–I).

Therefore, we selected these 4 miRNAs for the subsequent analysis. They all have the ability to bind with 4 genes out of the 6 coding genes ( Supplementary Figure S4C). Only 2 of these 6 genes could bind to these 4 miRNAs. The 2 genes are *CSF1* and *ICOSLG* ( Supplementary Figure S4D). To further confirm the 2 genes, we queried *CX3CL1* and their tissue expressions in NCBI and found wide expressions in various human tissues ( Supplementary Figure S7A–C). Next, we retrieved the expression of these 3 genes in the heart under single-cell conditions using the HPA database and found that they are expressed in all checked heart cell types, including cardiomyocytes, endothelial cells, immune cells, smooth muscle cells, and fibroblasts. The lowest expression was found in cardiomyocytes ( Supplementary Figure S7D–F). Published processes in NCBI confirmed their functions in inflammation/immunity-related processes ( Supplementary Figure S7G–I). To date, we identified the critical coding genes of our ceRNA network, including *CX3CL1*, *CSF1* and *ICOSLG*. Four miRNAs were annotated in InnatedDB, further confirming their immune/inflammatory roles ( Supplementary Figure S4E). Network analysis revealed that they can target various parts of the human heart, including muscle, artrium, atrial appendage, ventricle, and pericardium, which further confirmed the wide roles of these 4 miRNAs in the heart ( Supplementary Figure S4F). Thus, these 4 miRNAs were employed to constitute the ceRNA network. A total of 3 coding genes and 4 noncoding RNAs make up the ceRNA network. In short, the key components of our target ceRNA network screened by us are 3 coding genes, *CX3CL1*, *CSF1*, and *ICOSLG*, and 4 noncoding genes, hsa-miR-150-5p, hsa-miR-214-3p, hsa-miR-27a-3p, and hsa-miR-27b-3p ( Supplementary Figure S4G).

Our CX3CL1-circled network molecules are related to immune/inflammatory roles, so we want to dissect their relationships to ferroptosis, a recent interesting topic related to reactive oxygen species (ROS) balance. Three crucial signaling pathways of ferroptosis were analyzed. Eight genes, *SLC40A1* for Fe export; *TF*, *TFRC*, *SLC11A2*, and *LTF* for Fe uptake; *FTH1* and *FTL* for Fe storage; and *NCOA4* for ferritinophagy, were enlisted to parse associations with Fe homestasis. *ACSL4* and *LPCAT3* were analyzed for lipid peroxidation. The GPX4 axis consisting of SLC7A11, SLC3A2, CARS1, GCLC, GSS, and GPX4 was harnessed to address the inhibition of ferroptosis. We did not find any significant relationship between CX3CL1 and lipid peroxidation ( Figure 1A). CX3CL1 is positively related to LTF but negatively related to NCOA4. In the GPX4 axis, CX3CL1 is positively related to SLC3A2 and CARS1, two molecules with antagonistic effects on the production of CYS. These data suggest that CX3CL1 may regulate the internal balance of ferroptosis. ICOSLG is positively associated with ACSL4 for lipid peroxidation, TF, TFRC, SLC11A2, and LTF for Fe uptake, and FTH1 and FTL for Fe storage ( Figure 1B). ICOSLG is positively associated with SLC7A11, SLC3A2, and CARS1, which means that ICOSLG may have antagonistic effects on Cys quantities. In addition, ICOSLG is positively associated with GSS and GPX4. These data suggest that ICOSLG may also regulate ferroptosis internal balance. CSF1 is positively associated with ACSL4 for lipid peroxidation ( Figure 1C). For Fe homeostasis, CSF1 is positively associated with TF, TFRC, and SLC11A2 for Fe uptake; SLC40A1 for Fe export; and FTH1 and FTL for Fe storage. These results imply that CSF1 may regulate the internal balance of Fe homeostasis. CSF1 is positively associated with SLC7A11, SLC3A2, and CARS1, which means that CSF1 may have antagonistic effects on Cys quantities. Furthermore, CSF1 is positively associated with GSS and GPX4, showing that CSF1 may regulate ferroptosis internal balance. From the above analyses, we can see that our key network may modulate ferroptosis internal balance.

**Figure 1:**
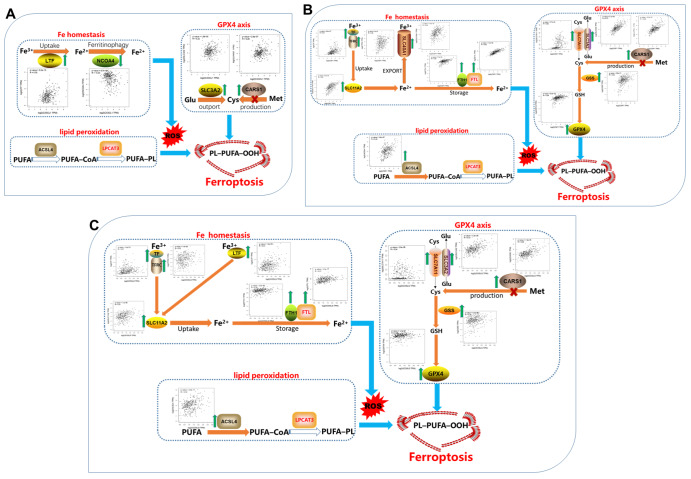
Figure 1 Signal map of correlations between CX3CL1-centered network molecules and key genes involved in ferroptosis (A) Signal map of correlations between CX3CL1 and key genes involved in ferroptosis. (B) Signal map of correlations between ICOSLG and key genes involved in ferroptosis. (C) Signal map of correlations between CSF1 and key genes involved in ferroptosis.

To further gain more functional information on the CX3CL1-circled network, we analyzed their relationships to macrophages in the heart. Conversion of macrophages from M1 to M2 is a critical event in immune/inflammatory modulation. We revealed that the M1 proportion has no significant relation to M2 ( Supplementary Figure S8A), while the M2 group is significantly higher than the M1 group ( Supplementary Figure S8B). Despite the large difference between the two groups, the absolute mean value was small. Next, we performed differential expression analysis for CX3CL1 in M1 and M2 cells and identified that CX3CL1 in M2 is significantly higher than that in M1 ( *P*<1×10 ^‒15^) and showed a very large F value (693.14) ( Supplementary Figure S8C). The same performance was observed for CSF1 (F=454.01) ( Supplementary Figure S8D) and ICOSLG (F=693.13) ( Supplementary Figure S8E). All these results suggest that the CX3CL1-mediated network may implicate the conversion of M1 macrophages to M2 macrophages or play an anti-inflammatory role.

From the above analyses, we established a molecular network consisting of three genes that have gone through all sorts of screening to act as mediators between the human heart and external stress. To confirm whether it is of clinical value, we went directly to clinical drug-related analysis. If clinical drug information cannot be found, the network is of little value. Luckily, we found much valuable drug information. First, we employed CTD, a robust publicly available database that can provide chemical-gene interactions for environmental exposures, to look for chemicals interplaying with the genes of our molecular network. Because the current database does not offer information on miRNAs, we only checked the three coding genes, *CX3CL1*, *CSF1* and *ICOSLG*. Ninety chemicals were found to target them ( Supplementary Figure S9A). Among them, 4 chemicals interact with *CSF1* and *ICOSLG*, 3 with *ICOSLG* and *CX3CL1*, 6 with *CX3CL1* and *CSF1*, and 5 with all three genes.

Then, we performed match analysis using DrugBank, an excellent clinical drug database, to verify whether some of these chemicals can be enlisted for use as drugs to treat clinical diseases. Critically, they must be in a position to treat not only inflammation/immunity-related diseases but also heart diseases. Only such drugs can confirm the important value of our molecular network having undergone all sorts of screening. Very excitingly, many such drugs have been screened from these chemicals. Top clinical trials of some of these drugs are shown here. Methotrexate had phase 3 for cardiovascular diseases, heart failure, and myocardial ischemia, phase 4 for inflammatory arthritis and phase 2 for COVID‑19 infections ( Supplementary Figure S9B). Arsenic trioxide showed phase 4 for acute promyelocytic leukemia (APL) and phase 2 for chronic graft versus host disease/immune system diseases ( Supplementary Figure S9C). Resveratrol had phase 4 for allergic rhinitis (disorder), phase 3 for acute pancreatitis, and phase 1 for heart failure ( Supplementary Figure S9D). Zoledronic acid possessed phase 4 for both complications of heart-lung transplant and HIV infections ( Supplementary Figure S9E). Cyclosporine had many clinical trials for heart diseases, for example, phase 3 for acute myocardial infarction (AMI) and phase 4 for disorders related to cardiac transplantation and for viral infections, including phase 4 for COVID-19 ( Supplementary Figure S9F). Epigallocatechin gallate had phase 2 for cardiac involvement, diabetic nephropathy, and diabetic nephropathy, and it also had clinical trials for tuberculosis (TB) and HIV infections ( Supplementary Figure S9G). Selenium saw phase 4 for cardiovascular disease (CVD), phase 3 for mycobacterium tuberculosis, and phase 1 for COVID‑19 infections ( Supplementary Figure S9H). Aspirin, a well-known drug, has been used in many clinical trials for various heart diseases in phase 4 and phase 2 for infection-related diseases, including COVID-19 infection ( Supplementary Figure S9I).

For a new ceRNA network, we believe that this proposed mechanism has to be experimentally explored in cellular and animal models to confirm its practical significance. Here, we resorted to another path to confirm this hypothesis by analyzing clinical drugs targeting this network. We identified 8 drugs in DrugBank ( Supplementary Figure S9B–I) from chemicals targeting our network ( Supplementary Figure S9A). Among them, 6 drugs ( Supplementary Figure S9B,D,F–I) have the potential to treat not only inflammation/immunity-related diseases but also heart diseases, which gives strong support to our idea. As we know, with its furtive asymptomatic transmissibility [ 6, 7], fighting COVID-19 is similar to fighting a series of large wars, so COVID-19 is also named WARS [ 8, 9]. Of note, 4 drugs may be enlisted to fight WARS ( Supplementary Figure S9B,F,H,I), indicating the value of our strategy in targeting inflammation/immunity-related pathways. Of interest, resveratrol ( Supplementary Figure S9D), epigallocatechin gallate ( Supplementary Figure S9G), and aspirin ( Supplementary Figure S9I) are all bitter medicines that can act as ligands of bitter taste receptors that belong to the TAS2R family, a subfamily of the GPCR family [ 10– 12]. Host-directed therapy by bitter medicine (HDT-BM) has been proposed to treat infection-related diseases, including WARS [ 13, 14], which naturally involves inflammatory/immune pathways. Therefore, HDT-BM may be a good strategy to treat heart disease coexisting with infection based on our network.

In summary, by employing public big data analysis, we finally established a new mechanism underlying the heart response to external stresses by the inflammatory/immune pathway mediated by the CX3CL1-centred network ( Figure 2). We found that CX3CL1 can form a ceRNA with CSF1 and ICOSLG via hsa-miR-150-5p, hsa-miR-214-3p, hsa-miR-27a-3p, or hsa-miR-27b-3p. This network may modulate the internal balance of ferroptosis homeostasis and macrophage function. We deciphered 6 clinical medicines from 90 chemicals targeting this inflammation/immunity-related ceRNA network, including the famous medicines aspirin, selenium, and resveratrol, which can be applied to treat both heart diseases and inflammation/immunity-related common diseases. Fascinatedly, methotrexate, cyclosporine, aspirin and selenium are used to treat COVID-19 in addition to heart diseases, which, to our surprise, roughs out a novel host-targeting antiviral scheme. Based on both basic and translational investigations, we assessed the value of the CX3CL1-formed ceRNA network for developing medicines to treat heart diseases, which provides important insights into heart diseases for basic research and drug development.

**Figure 2:**
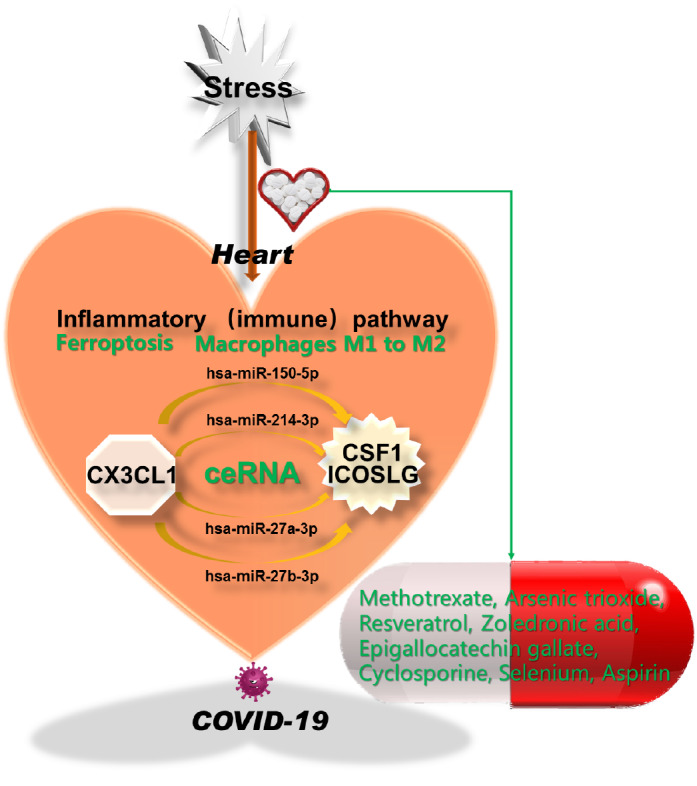
Figure 2 Diagram of the CX3CL1-centred network External stress/nonheart stress regulates heart functions or induces heart diseases through inflammatory or immune pathways mediated by the CX3CL1-centred network. Chemicals or drugs affect heart functions or treat heart diseases by targeting the ceRNA network consisting of 3 coding genes and 4 miRNAs.

## Supporting information

23332Supplementary_Data1

23332Supplementary-tables

23332supplementary-figures

## Supplementary Data

Supplementary data is available at *Biochimica et Biophysica Sinica* online.
