# Supplementary material for: Developing drugs targeting CX3CL1 to treat heart diseases via immune/inflammatory mediation: Targeting CX3CL1 to treat heart diseases
Source: Acta Biochim Biophys Sin (Shanghai). 2023 Aug 14;55(10):1672–6. doi: 10.3724/abbs.2023157 (PMC10577471; doi:10.3724/abbs.2023157)
Supplement: 23332Supplementary_Data1 [file 23332Supplementary_Data1.pdf]

Materials and Methods

Construction of coRNA and ceRNA geneset

GEPIA2 (gene expression profiling interactive analysis) dissects the gene expression and regulatory information of tens of thousands of tumor and non-tumor samples from TCGA and GTEx databases [1]. It was reclaimed to extract 200 of co-expression genes of CX3CL1 in heart. ENCORI (The Encyclopedia of RNA Interactomes) is a preeminent repository, which owns more than 2.5 million miRNA-mRNA interactions from CLIP-seq, degradome-seq and RNA-RNA interactome data and 10,882 RNA-seq and 10,546 miRNA-seq data on 32 types of tissues [2]. ceRNAs (competing endogenous RNAs) of CX3CL1 were examined by ENCORI and the target RNAs were extracted for further analysis. Online tool DAVID was enlisted to perform gene function annotation for co-expression dataset and ceRNA dataset of CX3CL1 [3]. Functional analyses were executed following the instructions of GOTERM-BP, GOTERM-CC, GOTERM-MF, KEGG-PATHWAY, HIV-INTERACTION (CATEGORY), and GAD-DISEASE (CLASS). The results were drawn by the brilliant tool IMAGEGP.

Extraction of intersection genes to construct ceRNA network

Another excellent tool EVENN was used to demonstrate all the intersection genes [4]. TissueAtlas, a web-based repository which harbors the expression data in 21 human organs using 188 samples, was used to profile the expression of miRNA in distinct tissues and draw network figure of miRNA in heart [5,6]. InnateDB is an online database for 2308 Innate Immunity Genes with detailed function annotations [7]. Functional annotations of intersection coding and miRNA genes were read from InnatedDB based on DAVID. DIANA-miRPath v3. 0 is an excellent online tool for deciphering microRNA function with experimental support [8]. It was enlisted to carry out function comments of KEGG pathway of the intersection miRNAs. Tissue profiling of intersection genes was downloaded from NCBI. The single cell expression analysis of intersection genes was performed in SCTA (Single Cell Type Atlas) in HPA (Human Protein Atlas) and SCTA has single cell RNA sequencing (scRNAseq) data from 13 distinct human tissues and includes all coding genes in 192 individual cell type clusters corresponding to 12 different cell type groups [9]. Functional processes of CX3CL1, CSF1 and ICOSLG were from NCBI and drawn

using IMAGEGP.

Dissection of the relations of ceRNA network with ferroptosis and macrophage

For parsing the involvement of CX3CL1-centred network in ferroptosis, we harnessed GTEx expression data in GEPIA to implement pair-wise gene correlation analysis. We utilized the correlation coefficient of spearman and the log-scale axis for visualization. For dissecting the relation of CX3CL1-centred network to macrophages, we selected the deconvolution tool of CIBERSORT to plot using heart expression data in GTEx data in GEPIA. Pearson correlation coefficient was dynamically calculated for macrophages M1 and M2.

Environmental exposures targeting the ceRNA network and drug match analysis

Comparative Toxicogenomics Database (CTD) is a comprehensive source database about effects of environmental exposures on human health [10]. Gene-chemical interactions were queried from CTD. DrugBank is a robust database which presents clinical information on drugs and drug targets [11]. They were used to do chemical matching analysis. The network of gene-chemical interplay was visualized by Cytoscape, a glistening drawing tool [12].

References

1. Tang Z, Kang B, Li C, Chen T, Zhang Z: **GEPIA2: an enhanced web server for large-scale expression profiling and interactive analysis**. *Nucleic Acids Res* 2019, **47**(W1):W556-W560.
2. Li JH, Liu S, Zhou H, Qu LH, Yang JH: **starBase v2.0: decoding miRNA-ceRNA, miRNA-ncRNA and protein-RNA interaction networks from large-scale CLIP-Seq data**. *Nucleic Acids Res* 2014, **42**(Database issue):D92-97.
3. Jiao X, Sherman BT, Huang da W, Stephens R, Baseler MW, Lane HC, Lempicki RA: **DAVID-WS: a stateful web service to facilitate gene/protein list analysis**. *Bioinformatics* 2012, **28**(13):1805-1806.
4. Chen T, Zhang H, Liu Y, Liu YX, Huang L: **EVenn: Easy to create repeatable and editable Venn diagrams and Venn networks online**. *J Genet Genomics* 2021, **48**(9):863-866.
5. Ludwig N, Leidinger P, Becker K, Backes C, Fehlmann T, Pallasch C,

Rheinheimer S, Meder B, Stahler C, Meese E *et al*: **Distribution of miRNA expression across human tissues**. *Nucleic Acids Res* 2016, **44**(8):3865-3877.

6. Fehlmann T, Ludwig N, Backes C, Meese E, Keller A: **Distribution of microRNA biomarker candidates in solid tissues and body fluids**. *RNA Biol* 2016, **13**(11):1084-1088.
7. Breuer K, Foroushani AK, Laird MR, Chen C, Sribnaia A, Lo R, Winsor GL, Hancock RE, Brinkman FS, Lynn DJ: **InnateDB: systems biology of innate immunity and beyond--recent updates and continuing curation**. *Nucleic Acids Res* 2013, **41**(Database issue):D1228-1233.
8. Vlachos IS, Zagganas K, Paraskevopoulou MD, Georgakilas G, Karagkouni D, Vergoulis T, Dalamagas T, Hatzigeorgiou AG: **DIANA-miRPath v3.0: deciphering microRNA function with experimental support**. *Nucleic Acids Res* 2015, **43**(W1):W460-466.
9. Uhlen M, Fagerberg L, Hallstrom BM, Lindskog C, Oksvold P, Mardinoglu A, Sivertsson A, Kampf C, Sjostedt E, Asplund A *et al*: **Proteomics. Tissue-based map of the human proteome**. *Science* 2015, **347**(6220):1260419.
10. Davis AP, Grondin CJ, Johnson RJ, Sciaky D, Wiegers J, Wiegers TC, Mattingly CJ: **Comparative Toxicogenomics Database (CTD): update 2021**. *Nucleic Acids Res* 2021, **49**(D1):D1138-D1143.
11. Wishart DS, Feunang YD, Guo AC, Lo EJ, Marcu A, Grant JR, Sajed T, Johnson D, Li C, Sayeeda Z *et al*: **DrugBank 5.0: a major update to the DrugBank database for 2018**. *Nucleic Acids Res* 2018, **46**(D1):D1074-D1082.
12. Shannon P, Markiel A, Ozier O, Baliga NS, Wang JT, Ramage D, Amin N, Schwikowski B, Ideker T: **Cytoscape: a software environment for integrated models of biomolecular interaction networks**. *Genome Res* 2003, **13**(11):2498-2504.
