# Supplementary material for: Developing drugs targeting CX3CL1 to treat heart diseases via immune/inflammatory mediation: Targeting CX3CL1 to treat heart diseases
Source: Acta Biochim Biophys Sin (Shanghai). 2023 Aug 14;55(10):1672–6. doi: 10.3724/abbs.2023157 (PMC10577471; doi:10.3724/abbs.2023157)
Supplement: 23332supplementary-figures [file 23332supplementary-figures.pdf]

FIG.S1

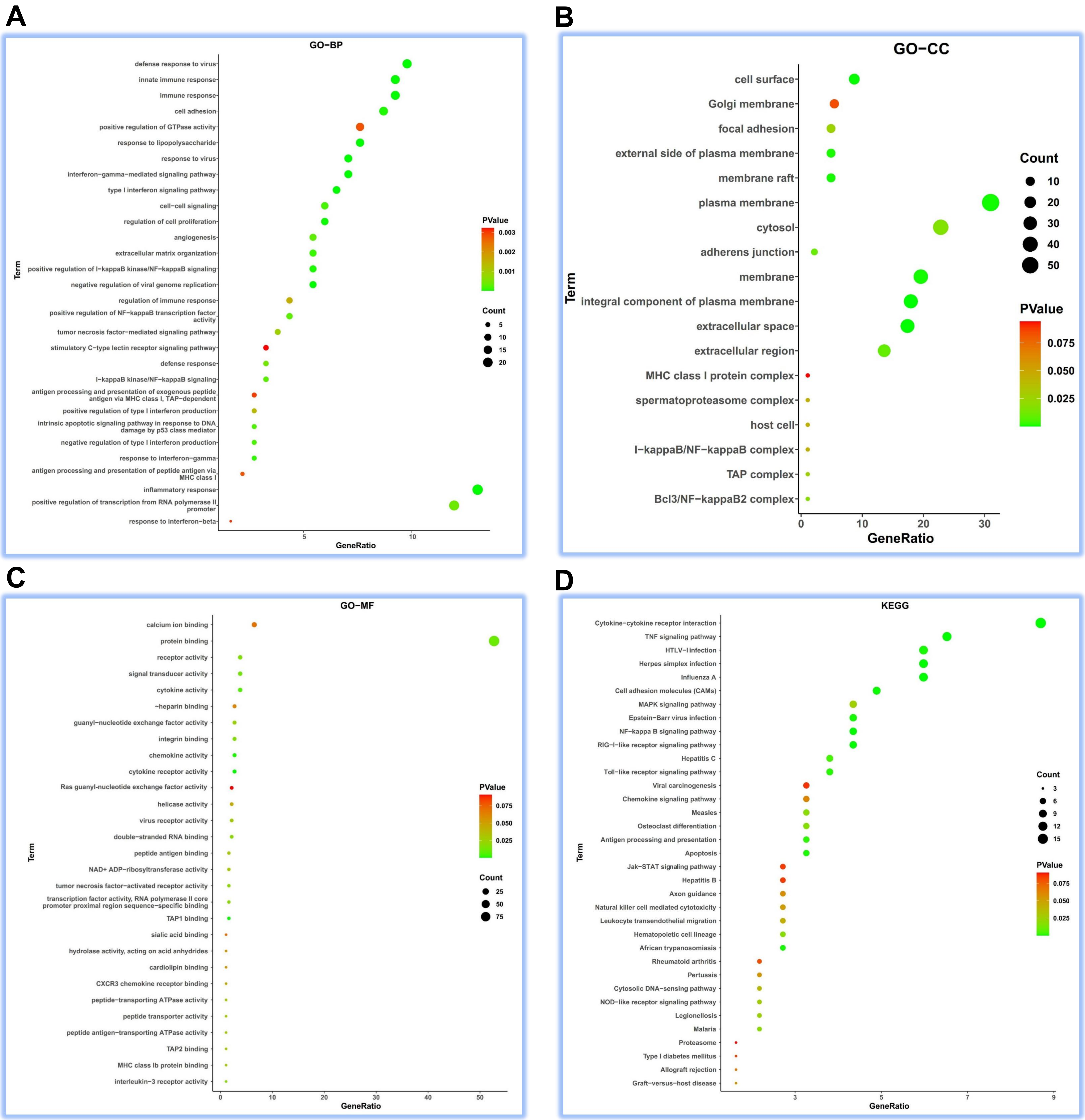
A
GO−BP
defense response to virus
innate immune response
immune response
cell adhesion
positive regulation of GTPase activity
response to lipopolysaccharide
response to virus
interferon−gamma−mediated signaling pathway
type I interferon signaling pathway
cell−cell signaling
regulation of cell proliferation
angiogenesis
extracellular matrix organization
positive regulation of I−kappaB kinase/NF−kappaB signaling
negative regulation of viral genome replication
regulation of immune response
positive regulation of NF−kappaB transcription factor activity
tumor necrosis factor−mediated signaling pathway
stimulatory C−type lectin receptor signaling pathway
defense response
I−kappaB kinase/NF−kappaB signaling
antigen processing and presentation of exogenous peptide antigen via MHC class I, TAP−dependent
positive regulation of type I interferon production
intrinsic apoptotic signaling pathway in response to DNA damage by p53 class mediator
negative regulation of type I interferon production
response to interferon−gamma
antigen processing and presentation of peptide antigen via MHC class I
inflammatory response
positive regulation of transcription from RNA polymerase II promoter
response to interferon−beta
Term
GeneRatio
5
10
PValue
0.003
0.002
0.001
Count
5
10
15
20
B
GO−CC
cell surface
Golgi membrane
focal adhesion
external side of plasma membrane
membrane raft
plasma membrane
cytosol
adherens junction
membrane
integral component of plasma membrane
extracellular space
extracellular region
MHC class I protein complex
spermatoproteasome complex
host cell
I−kappaB/NF−kappaB complex
TAP complex
Bcl3/NF−kappaB2 complex
Term
GeneRatio
0
10
20
30
Count
10
20
30
40
50
PValue
0.075
0.050
0.025
C
GO−MF
calcium ion binding
protein binding
receptor activity
signal transducer activity
cytokine activity
~heparin binding
guanyl−nucleotide exchange factor activity
integrin binding
chemokine activity
cytokine receptor activity
Ras guanyl−nucleotide exchange factor activity
helicase activity
virus receptor activity
double−stranded RNA binding
peptide antigen binding
NAD+ ADP−ribosyltransferase activity
tumor necrosis factor−activated receptor activity
transcription factor activity, RNA polymerase II core promoter proximal region sequence−specific binding
TAP1 binding
sialic acid binding
hydrolase activity, acting on acid anhydrides
cardiolipin binding
CXCR3 chemokine receptor binding
peptide−transporting ATPase activity
peptide transporter activity
peptide antigen−transporting ATPase activity
TAP2 binding
MHC class Ib protein binding
interleukin−3 receptor activity
Term
GeneRatio
0
10
20
30
40
50
PValue
0.075
0.050
0.025
Count
25
50
75
D
KEGG
Cytokine−cytokine receptor interaction
TNF signaling pathway
HTLV−I infection
Herpes simplex infection
Influenza A
Cell adhesion molecules (CAMs)
MAPK signaling pathway
Epstein−Barr virus infection
NF−kappa B signaling pathway
RIG−I−like receptor signaling pathway
Hepatitis C
Toll−like receptor signaling pathway
Viral carcinogenesis
Chemokine signaling pathway
Measles
Osteoclast differentiation
Antigen processing and presentation
Apoptosis
Jak−STAT signaling pathway
Hepatitis B
Axon guidance
Natural killer cell mediated cytotoxicity
Leukocyte transendothelial migration
Hematopoietic cell lineage
African trypanosomiasis
Rheumatoid arthritis
Pertussis
Cytosolic DNA−sensing pathway
NOD−like receptor signaling pathway
Legionellosis
Malaria
Proteasome
Type I diabetes mellitus
Allograft rejection
Graft−versus−host disease
Term
GeneRatio
3
5
7
9
Count
3
6
9
12
15
PValue
0.075
0.050
0.025

E

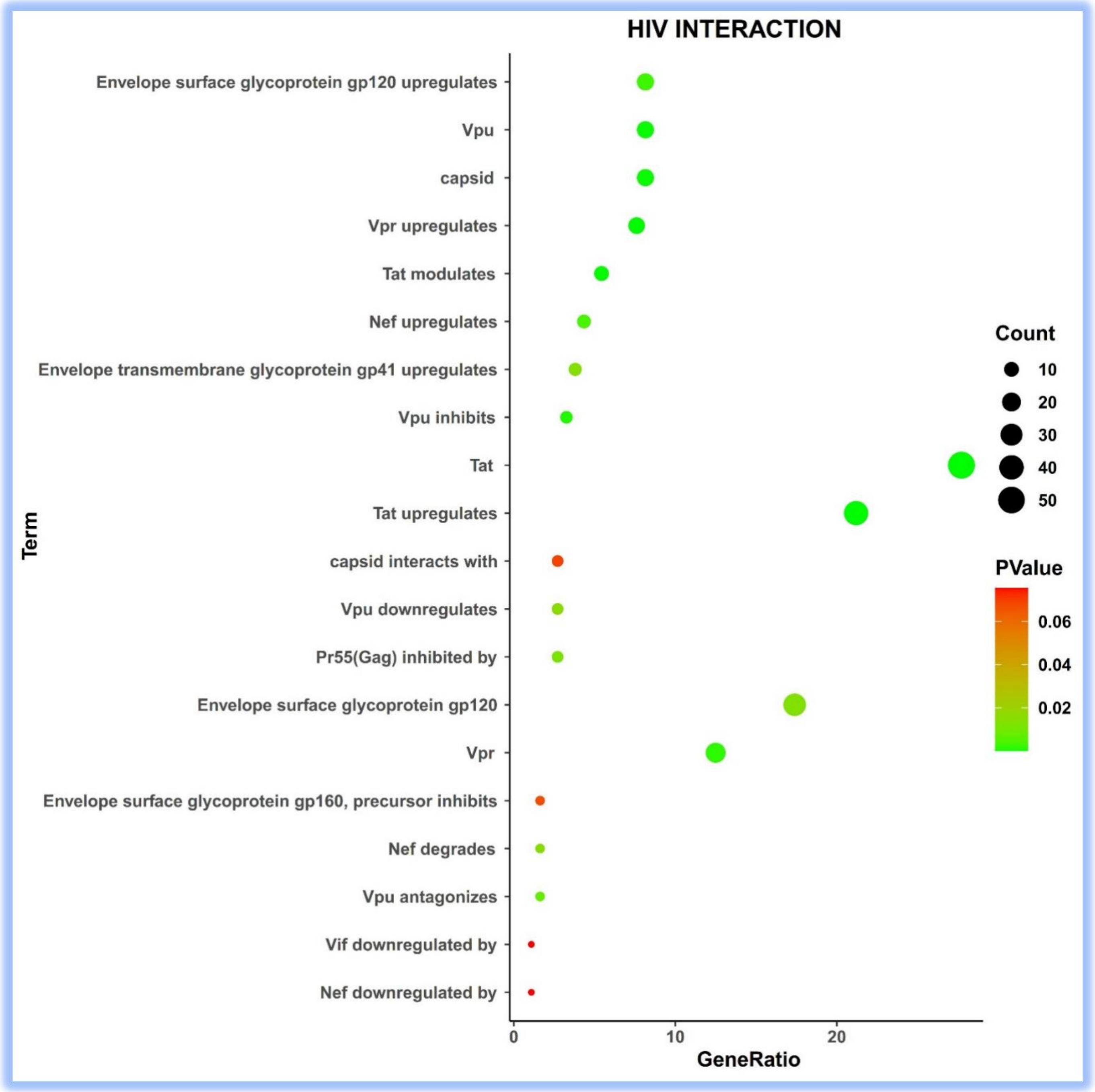

HIV INTERACTION
Envelope surface glycoprotein gp120 upregulates
Vpu
capsid
Vpr upregulates
Tat modulates
Nef upregulates
Envelope transmembrane glycoprotein gp41 upregulates
Vpu inhibits
Tat
Tat upregulates
capsid interacts with
Vpu downregulates
Pr55(Gag) inhibited by
Envelope surface glycoprotein gp120
Vpr
Envelope surface glycoprotein gp160, precursor inhibits
Nef degrades
Vpu antagonizes
Vif downregulated by
Nef downregulated by
Term
0
10
20
GeneRatio
Count
10
20
30
40
50
PValue
0.06
0.04
0.02

F

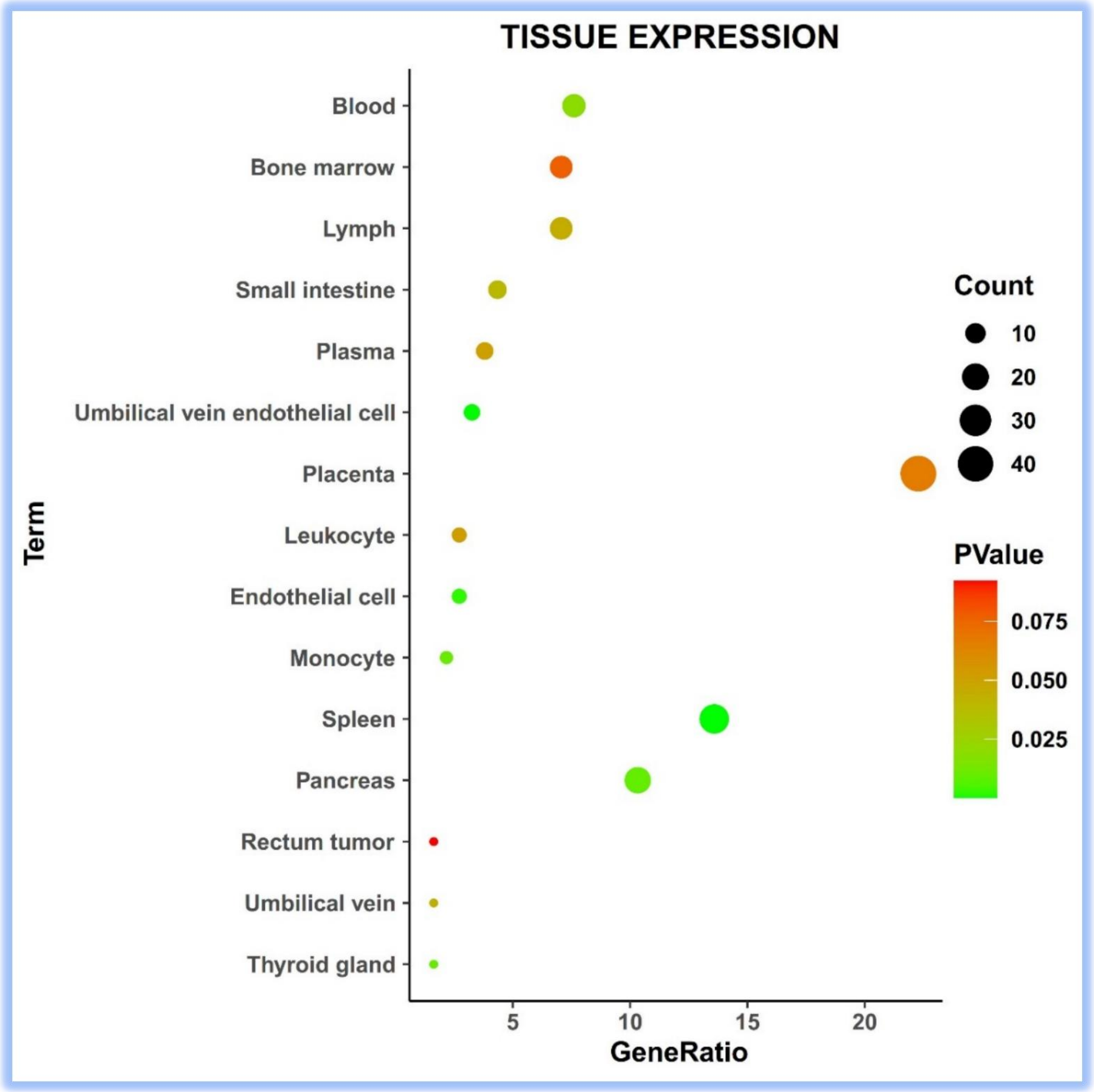

TISSUE EXPRESSION
Blood
Bone marrow
Lymph
Small intestine
Plasma
Umbilical vein endothelial cell
Placenta
Leukocyte
Endothelial cell
Monocyte
Spleen
Pancreas
Rectum tumor
Umbilical vein
Thyroid gland
Term
5
10
15
20
GeneRatio
Count
10
20
30
40
PValue
0.075
0.050
0.025

G

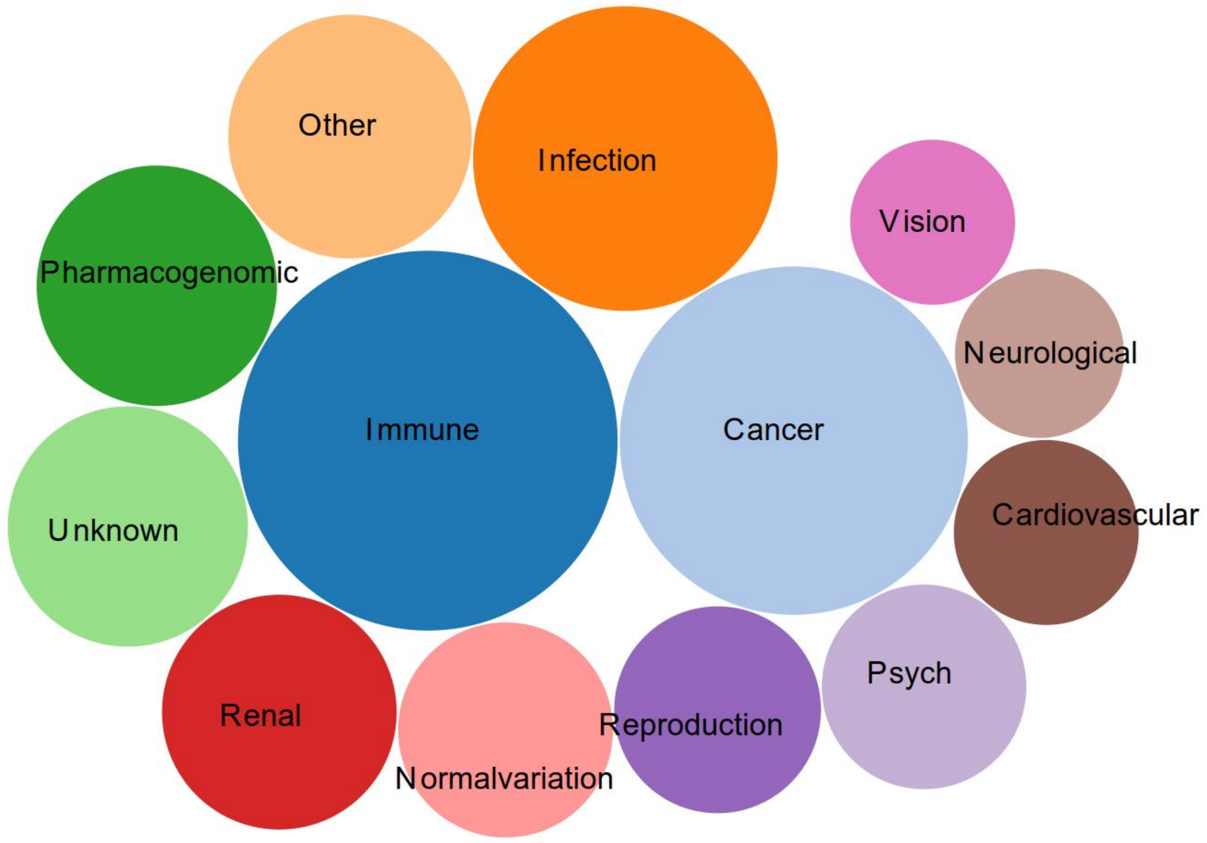

Other
Infection
Vision
Pharmacogenomic
Neurological
Immune
Cancer
Unknown
Cardiovascular
Psych
Renal
Reproduction
Normalvariation

Supplementary Figure S1. Extensively parse functional terms of CX3CL1 in heart by gene co-expression analysis A total of 200 co-expression genes of CX3CL1 in human heart were extracted. The analysis of GO, KEGG pathway, HIV interaction, tissue distribution and GAD DISEASE based on these genes was performed by DAVID tool. CX3CL1 has a wide range of functions, obviously enriched in the inflammation or immune-related function in heart. (A) Functional terms by GO-BP analysis. (B) Functional terms by GO-CC analysis. (C) Functional terms by GO-MF analysis. (D) Functional terms by KEGG analysis. (E) Functional terms by HIV interaction analysis. (F) Functional terms by tissue expression analysis. (G) Functional terms by GAD DISEASE analysis. The size of the circles indicates a negative Log10 (*P* value). GO, Gene Ontology; BP, biological process; CC, cellular component; MF, molecular function.

FIG.S2

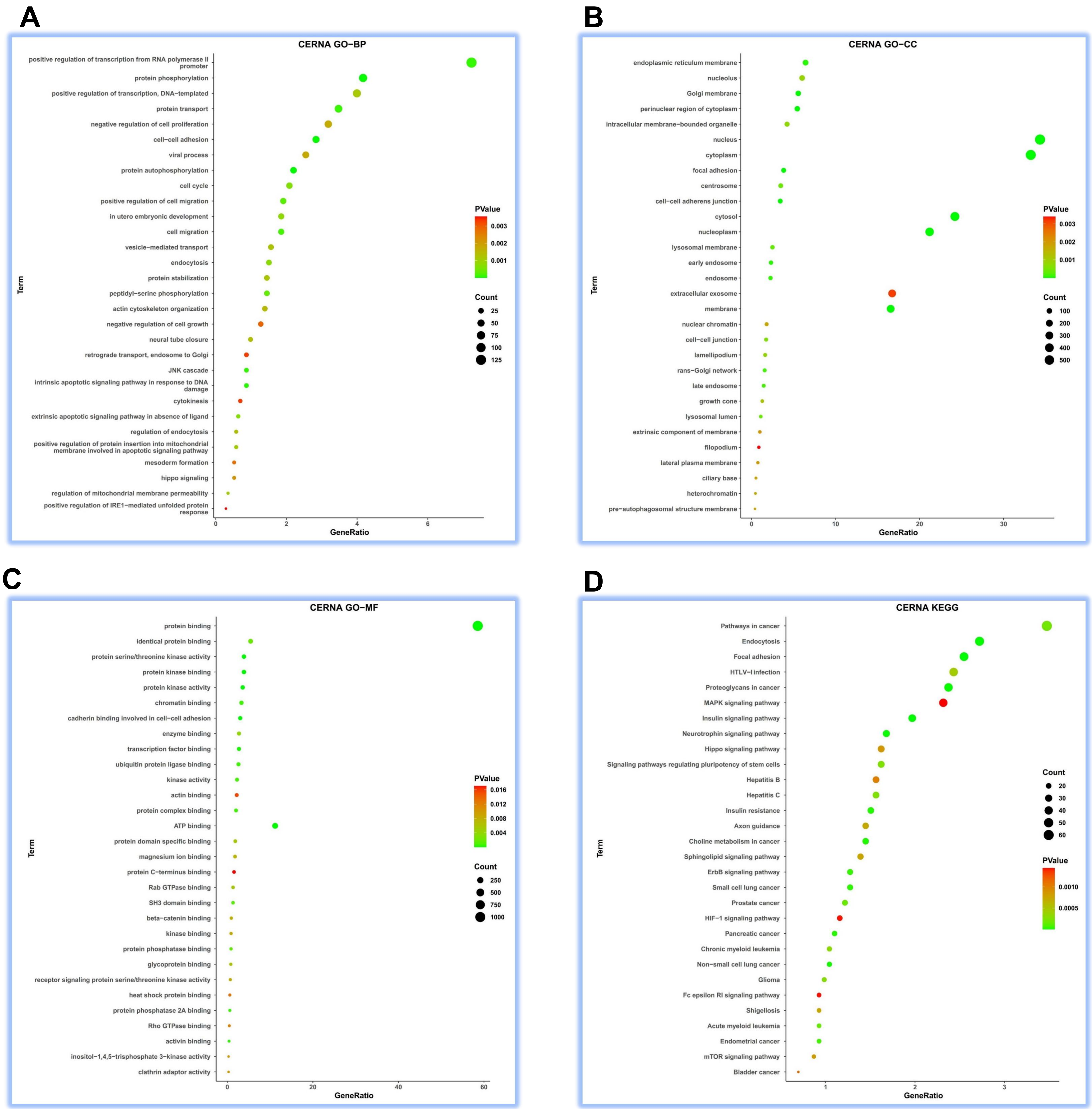
A
CERNA GO−BP
positive regulation of transcription from RNA polymerase II promoter
protein phosphorylation
positive regulation of transcription, DNA−templated
protein transport
negative regulation of cell proliferation
cell−cell adhesion
viral process
protein autophosphorylation
cell cycle
positive regulation of cell migration
in utero embryonic development
cell migration
vesicle−mediated transport
endocytosis
protein stabilization
peptidyl−serine phosphorylation
actin cytoskeleton organization
negative regulation of cell growth
neural tube closure
retrograde transport, endosome to Golgi
JNK cascade
intrinsic apoptotic signaling pathway in response to DNA damage
cytokinesis
extrinsic apoptotic signaling pathway in absence of ligand
regulation of endocytosis
positive regulation of protein insertion into mitochondrial membrane involved in apoptotic signaling pathway
mesoderm formation
hippo signaling
regulation of mitochondrial membrane permeability
positive regulation of IRE1−mediated unfolded protein response
Term
GeneRatio
0
2
4
6
PValue
0.003
0.002
0.001
Count
25
50
75
100
125
B
CERNA GO−CC
endoplasmic reticulum membrane
nucleolus
Golgi membrane
perinuclear region of cytoplasm
intracellular membrane−bounded organelle
nucleus
cytoplasm
focal adhesion
centrosome
cell−cell adherens junction
cytosol
nucleoplasm
lysosomal membrane
early endosome
endosome
extracellular exosome
membrane
nuclear chromatin
cell−cell junction
lamellipodium
rans−Golgi network
late endosome
growth cone
lysosomal lumen
extrinsic component of membrane
filopodium
lateral plasma membrane
ciliary base
heterochromatin
pre−autophagosomal structure membrane
Term
GeneRatio
0
10
20
30
PValue
0.003
0.002
0.001
Count
100
200
300
400
500
C
CERNA GO−MF
protein binding
identical protein binding
protein serine/threonine kinase activity
protein kinase binding
protein kinase activity
chromatin binding
cadherin binding involved in cell−cell adhesion
enzyme binding
transcription factor binding
ubiquitin protein ligase binding
kinase activity
actin binding
protein complex binding
ATP binding
protein domain specific binding
magnesium ion binding
protein C−terminus binding
Rab GTPase binding
SH3 domain binding
beta−catenin binding
kinase binding
protein phosphatase binding
glycoprotein binding
receptor signaling protein serine/threonine kinase activity
heat shock protein binding
protein phosphatase 2A binding
Rho GTPase binding
activin binding
inositol−1,4,5−trisphosphate 3−kinase activity
clathrin adaptor activity
Term
GeneRatio
0
20
40
60
PValue
0.016
0.012
0.008
0.004
Count
250
500
750
1000
D
CERNA KEGG
Pathways in cancer
Endocytosis
Focal adhesion
HTLV−I infection
Proteoglycans in cancer
MAPK signaling pathway
Insulin signaling pathway
Neurotrophin signaling pathway
Hippo signaling pathway
Signaling pathways regulating pluripotency of stem cells
Hepatitis B
Hepatitis C
Insulin resistance
Axon guidance
Choline metabolism in cancer
Sphingolipid signaling pathway
ErbB signaling pathway
Small cell lung cancer
Prostate cancer
HIF−1 signaling pathway
Pancreatic cancer
Chronic myeloid leukemia
Non−small cell lung cancer
Glioma
Fc epsilon RI signaling pathway
Shigellosis
Acute myeloid leukemia
Endometrial cancer
mTOR signaling pathway
Bladder cancer
Term
GeneRatio
1
2
3
Count
20
30
40
50
60
PValue
0.0010
0.0005

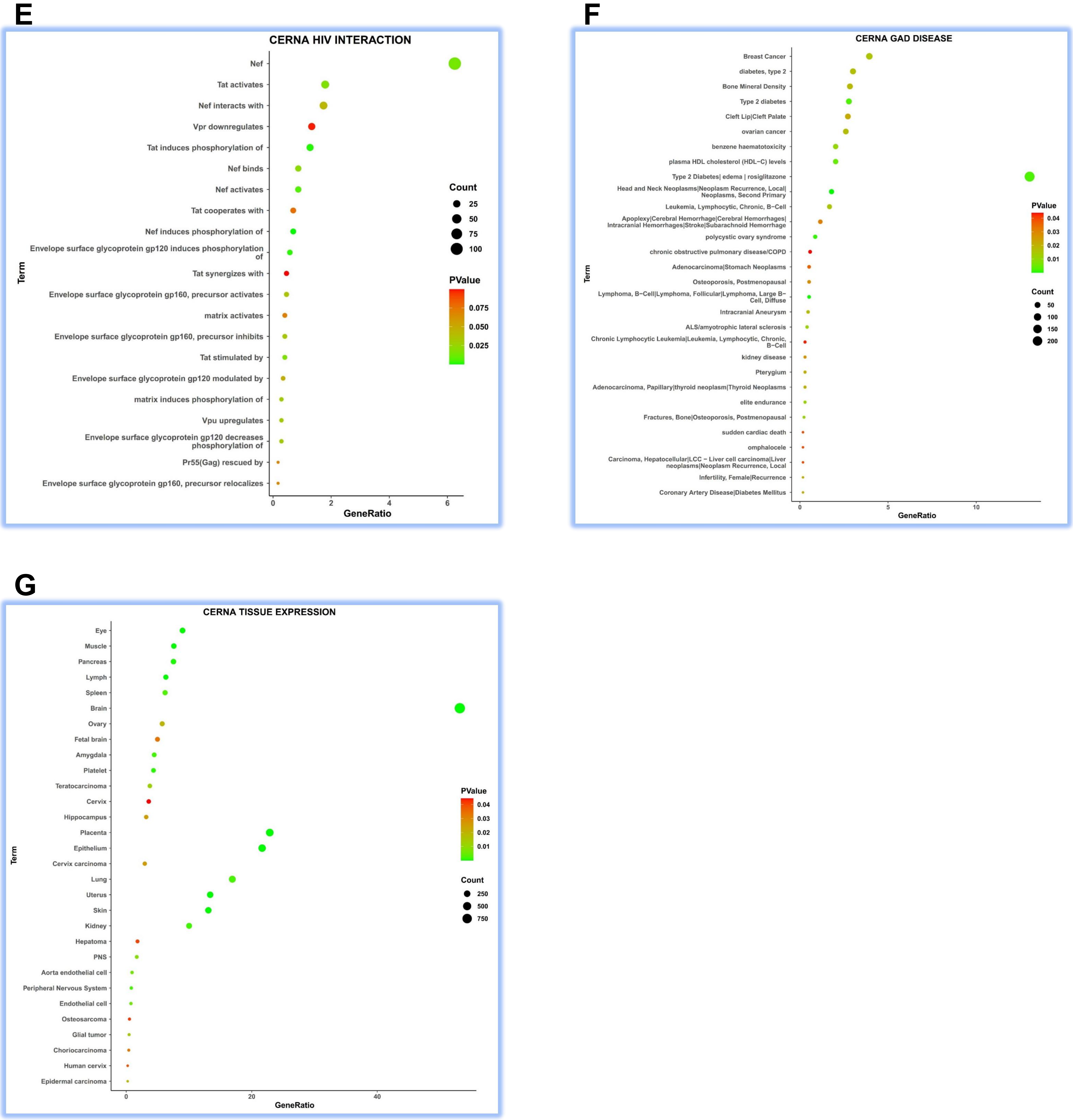
E
CERNA HIV INTERACTION
Nef
Tat activates
Nef interacts with
Vpr downregulates
Tat induces phosphorylation of
Nef binds
Nef activates
Tat cooperates with
Nef induces phosphorylation of
Envelope surface glycoprotein gp120 induces phosphorylation of
Tat synergizes with
Envelope surface glycoprotein gp160, precursor activates
matrix activates
Envelope surface glycoprotein gp160, precursor inhibits
Tat stimulated by
Envelope surface glycoprotein gp120 modulated by
matrix induces phosphorylation of
Vpu upregulates
Envelope surface glycoprotein gp120 decreases phosphorylation of
Pr55(Gag) rescued by
Envelope surface glycoprotein gp160, precursor relocalizes
Term
GeneRatio
Count
PValue
F
CERNA GAD DISEASE
Breast Cancer
diabetes, type 2
Bone Mineral Density
Type 2 diabetes
Cleft Lip|Cleft Palate
ovarian cancer
benzene haematotoxicity
plasma HDL cholesterol (HDL−C) levels
Type 2 Diabetes| edema | rosiglitazone
Head and Neck Neoplasms|Neoplasm Recurrence, Local|Neoplasms, Second Primary
Leukemia, Lymphocytic, Chronic, B−Cell
Apoplexy|Cerebral Hemorrhage|Cerebral Hemorrhages|Intracranial Hemorrhages|Stroke|Subarachnoid Hemorrhage
polycystic ovary syndrome
chronic obstructive pulmonary disease/COPD
Adenocarcinoma|Stomach Neoplasms
Osteoporosis, Postmenopausal
Lymphoma, B−Cell|Lymphoma, Follicular|Lymphoma, Large B−Cell, Diffuse
Intracranial Aneurysm
ALS/amyotrophic lateral sclerosis
Chronic Lymphocytic Leukemia|Leukemia, Lymphocytic, Chronic, B−Cell
kidney disease
Pterygium
Adenocarcinoma, Papillary|thyroid neoplasm|Thyroid Neoplasms
elite endurance
Fractures, Bone|Osteoporosis, Postmenopausal
sudden cardiac death
omphalocele
Carcinoma, Hepatocellular|LCC − Liver cell carcinoma|Liver neoplasms|Neoplasm Recurrence, Local
Infertility, Female|Recurrence
Coronary Artery Disease|Diabetes Mellitus
Term
GeneRatio
PValue
Count
G
CERNA TISSUE EXPRESSION
Eye
Muscle
Pancreas
Lymph
Spleen
Brain
Ovary
Fetal brain
Amygdala
Platelet
Teratocarcinoma
Cervix
Hippocampus
Placenta
Epithelium
Cervix carcinoma
Lung
Uterus
Skin
Kidney
Hepatoma
PNS
Aorta endothelial cell
Peripheral Nervous System
Endothelial cell
Osteosarcoma
Glial tumor
Choriocarcinoma
Human cervix
Epidermal carcinoma
Term
GeneRatio
PValue
Count

Supplementary Figure S2. Dissection of extensive functional annotations of CX3CL1 in human tissues outside the heart by ceRNA analysis A total of 1782 genes sharing ceRNA mechanism with CX3CL1 in 32 human tissues were extracted. DAVID tool was enlisted to analyze these genes on Gene Ontology (GO), KEGG pathway, HIV interaction, tissue distribution and GAD. CX3CL1 holds a wide cellular and tissue expression and diverse functions. (A) Functional terms by GO-BP analysis. (B) Functional terms by GO-CC analysis. (C) Functional terms by GO-MF analysis. (D) Functional terms by KEGG analysis. (E) Functional terms by HIV interaction analysis. (F) Functional terms by GAD DISEASE analysis. (G) Functional terms by tissue expression analysis GO, Gene Ontology; BP, biological process; CC, cellular component; MF, molecular function.

FIG.S3

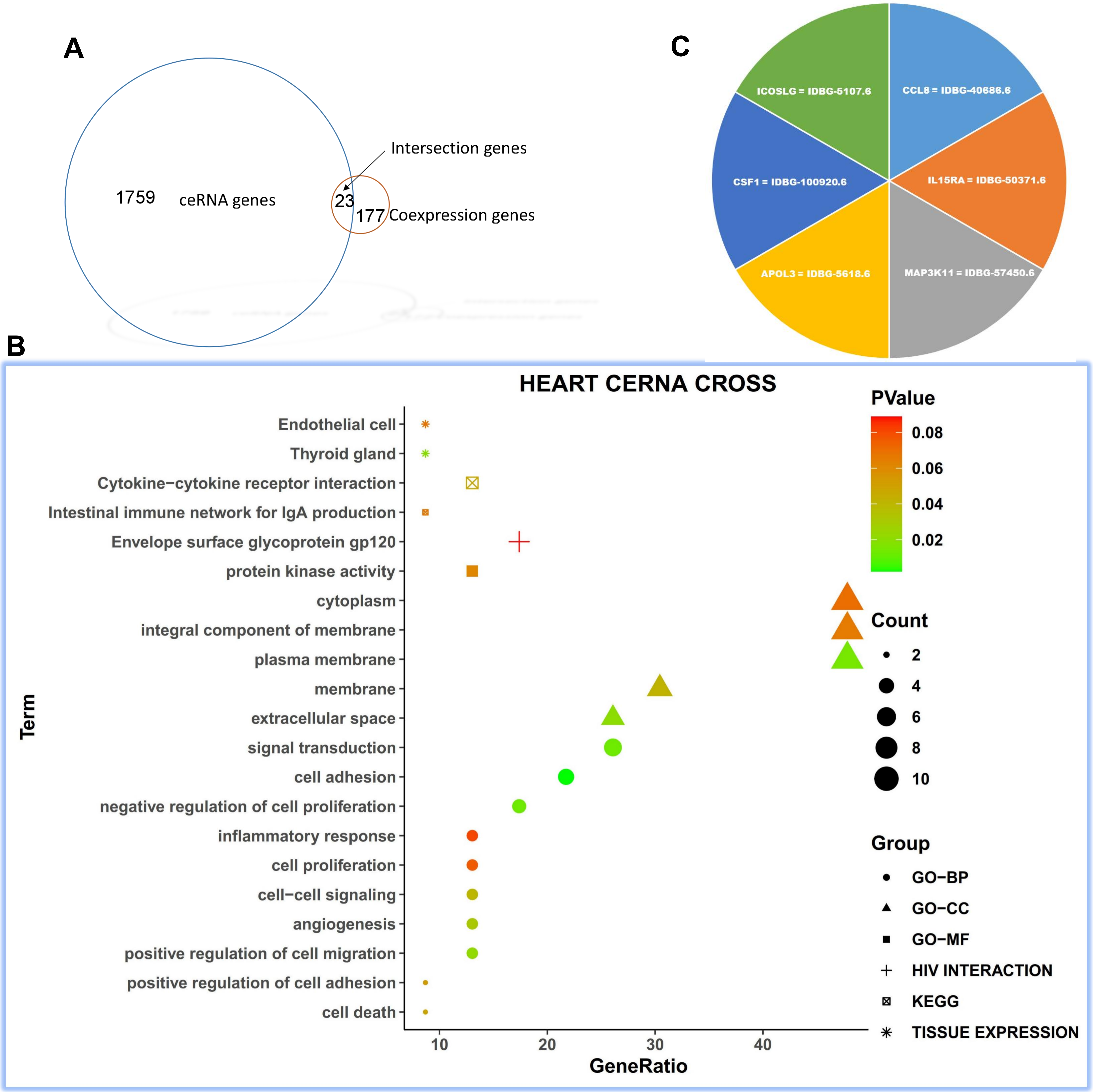
A
1759
ceRNA genes
23
177
Intersection genes
Coexpression genes
C
CCL8 = IDBG-40686.6
IL15RA = IDBG-50371.6
MAP3K11 = IDBG-57450.6
APOL3 = IDBG-5618.6
CSF1 = IDBG-100920.6
ICOSLG = IDBG-5107.6
B
HEART CERNA CROSS
Endothelial cell
Thyroid gland
Cytokine−cytokine receptor interaction
Intestinal immune network for IgA production
Envelope surface glycoprotein gp120
protein kinase activity
cytoplasm
integral component of membrane
plasma membrane
membrane
extracellular space
signal transduction
cell adhesion
negative regulation of cell proliferation
inflammatory response
cell proliferation
cell−cell signaling
angiogenesis
positive regulation of cell migration
positive regulation of cell adhesion
cell death
Term
10
20
30
40
GeneRatio
PValue
0.08
0.06
0.04
0.02
Count
2
4
6
8
10
Group
GO−BP
GO−CC
GO−MF
HIV INTERACTION
KEGG
TISSUE EXPRESSION

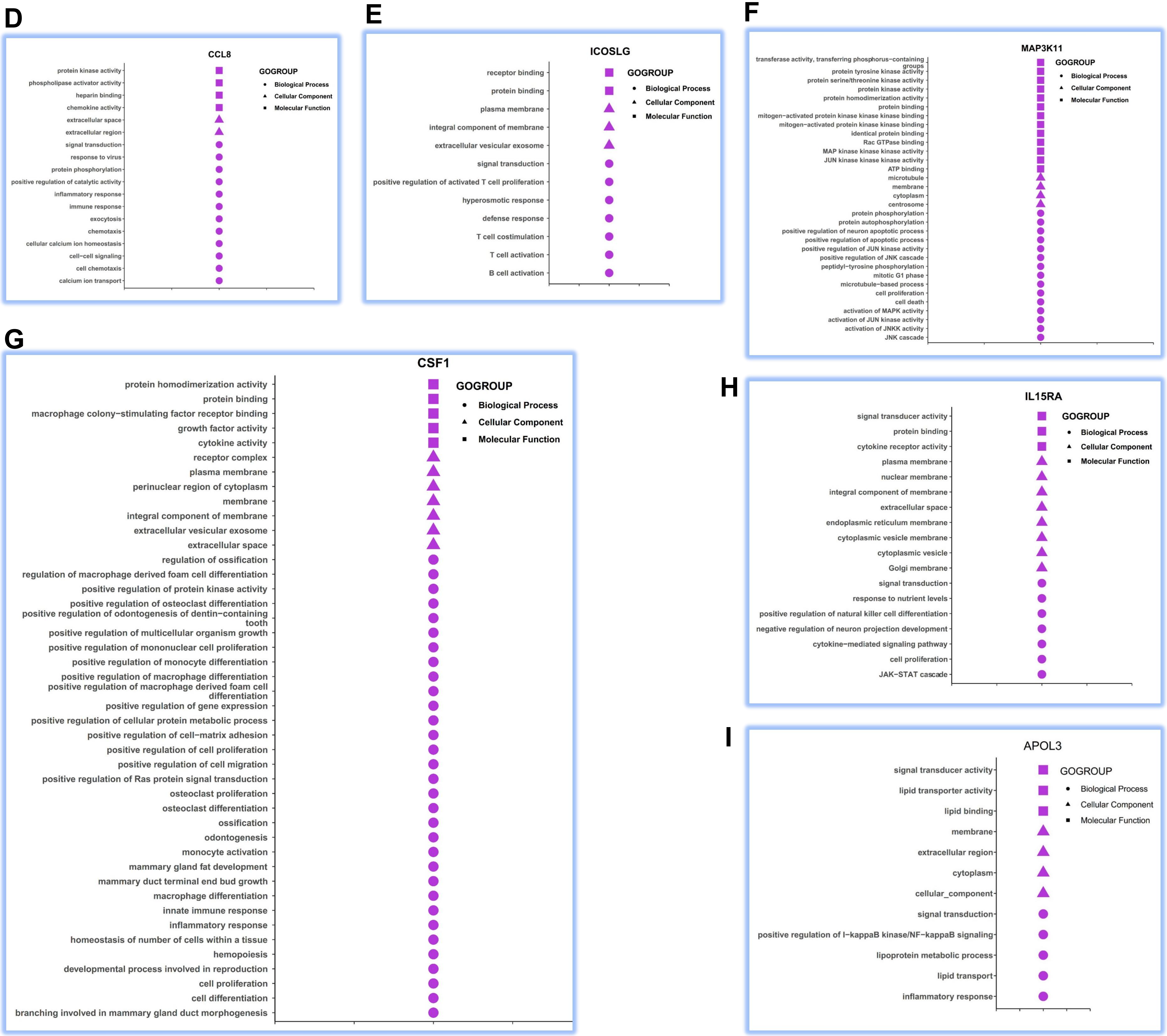
D
CCL8
protein kinase activity
phospholipase activator activity
heparin binding
chemokine activity
extracellular space
extracellular region
signal transduction
response to virus
protein phosphorylation
positive regulation of catalytic activity
inflammatory response
immune response
exocytosis
chemotaxis
cellular calcium ion homeostasis
cell−cell signaling
cell chemotaxis
calcium ion transport
GOGROUP
Biological Process
Cellular Component
Molecular Function
E
ICOSLG
receptor binding
protein binding
plasma membrane
integral component of membrane
extracellular vesicular exosome
signal transduction
positive regulation of activated T cell proliferation
hyperosmotic response
defense response
T cell costimulation
T cell activation
B cell activation
GOGROUP
Biological Process
Cellular Component
Molecular Function
F
MAP3K11
transferase activity, transferring phosphorus−containing groups
protein tyrosine kinase activity
protein serine/threonine kinase activity
protein kinase activity
protein homodimerization activity
protein binding
mitogen−activated protein kinase kinase kinase binding
mitogen−activated protein kinase kinase binding
identical protein binding
Rac GTPase binding
MAP kinase kinase kinase activity
JUN kinase kinase kinase activity
ATP binding
microtubule
membrane
cytoplasm
centrosome
protein phosphorylation
protein autophosphorylation
positive regulation of neuron apoptotic process
positive regulation of apoptotic process
positive regulation of JUN kinase activity
positive regulation of JNK cascade
peptidyl−tyrosine phosphorylation
mitotic G1 phase
microtubule−based process
cell proliferation
cell death
activation of MAPK activity
activation of JUN kinase activity
activation of JNKK activity
JNK cascade
GOGROUP
Biological Process
Cellular Component
Molecular Function
G
CSF1
protein homodimerization activity
protein binding
macrophage colony−stimulating factor receptor binding
growth factor activity
cytokine activity
receptor complex
plasma membrane
perinuclear region of cytoplasm
membrane
integral component of membrane
extracellular vesicular exosome
extracellular space
regulation of ossification
regulation of macrophage derived foam cell differentiation
positive regulation of protein kinase activity
positive regulation of osteoclast differentiation
positive regulation of odontogenesis of dentin−containing tooth
positive regulation of multicellular organism growth
positive regulation of mononuclear cell proliferation
positive regulation of monocyte differentiation
positive regulation of macrophage differentiation
positive regulation of macrophage derived foam cell differentiation
positive regulation of gene expression
positive regulation of cellular protein metabolic process
positive regulation of cell−matrix adhesion
positive regulation of cell proliferation
positive regulation of cell migration
positive regulation of Ras protein signal transduction
osteoclast proliferation
osteoclast differentiation
ossification
odontogenesis
monocyte activation
mammary gland fat development
mammary duct terminal end bud growth
macrophage differentiation
innate immune response
inflammatory response
homeostasis of number of cells within a tissue
hemopoiesis
developmental process involved in reproduction
cell proliferation
cell differentiation
branching involved in mammary gland duct morphogenesis
GOGROUP
Biological Process
Cellular Component
Molecular Function
H
IL15RA
signal transducer activity
protein binding
cytokine receptor activity
plasma membrane
nuclear membrane
integral component of membrane
extracellular space
endoplasmic reticulum membrane
cytoplasmic vesicle membrane
cytoplasmic vesicle
Golgi membrane
signal transduction
response to nutrient levels
positive regulation of natural killer cell differentiation
negative regulation of neuron projection development
cytokine−mediated signaling pathway
cell proliferation
JAK−STAT cascade
GOGROUP
Biological Process
Cellular Component
Molecular Function
I
APOL3
signal transducer activity
lipid transporter activity
lipid binding
membrane
extracellular region
cytoplasm
cellular_component
signal transduction
positive regulation of I−kappaB kinase/NF−kappaB signaling
lipoprotein metabolic process
lipid transport
inflammatory response
GOGROUP
Biological Process
Cellular Component
Molecular Function

Supplementary Figure S3. Twenty-three sharing genes were found in ceRNA and co-expression genes of CX3CL1 Functional annotations were analyzed by DAVID. And 6 immune or inflammation-related cross genes were extracted to query in NCBI to look for notes of Gene Ontology. (A) Gene numbers of distinct datasets. (B) Function terms of 23 cross genes by DAVID. (C) Six intersection genes were annotated in innateDB. (D) GO terms of CCL8 queried in NCBI. (E) GO terms of ICOSLG queried in NCBI. (F) GO terms of MAP3K11 queried in NCBI. (G) GO terms of CSF1 queried in NCBI. (H) GO terms of IL15RA queried in NCBI. (I) GO terms of APOL3 queried in NCBI. GO, Gene Ontology; BP, biological process; CC, cellular component; MF, molecular function.

FIG.S4

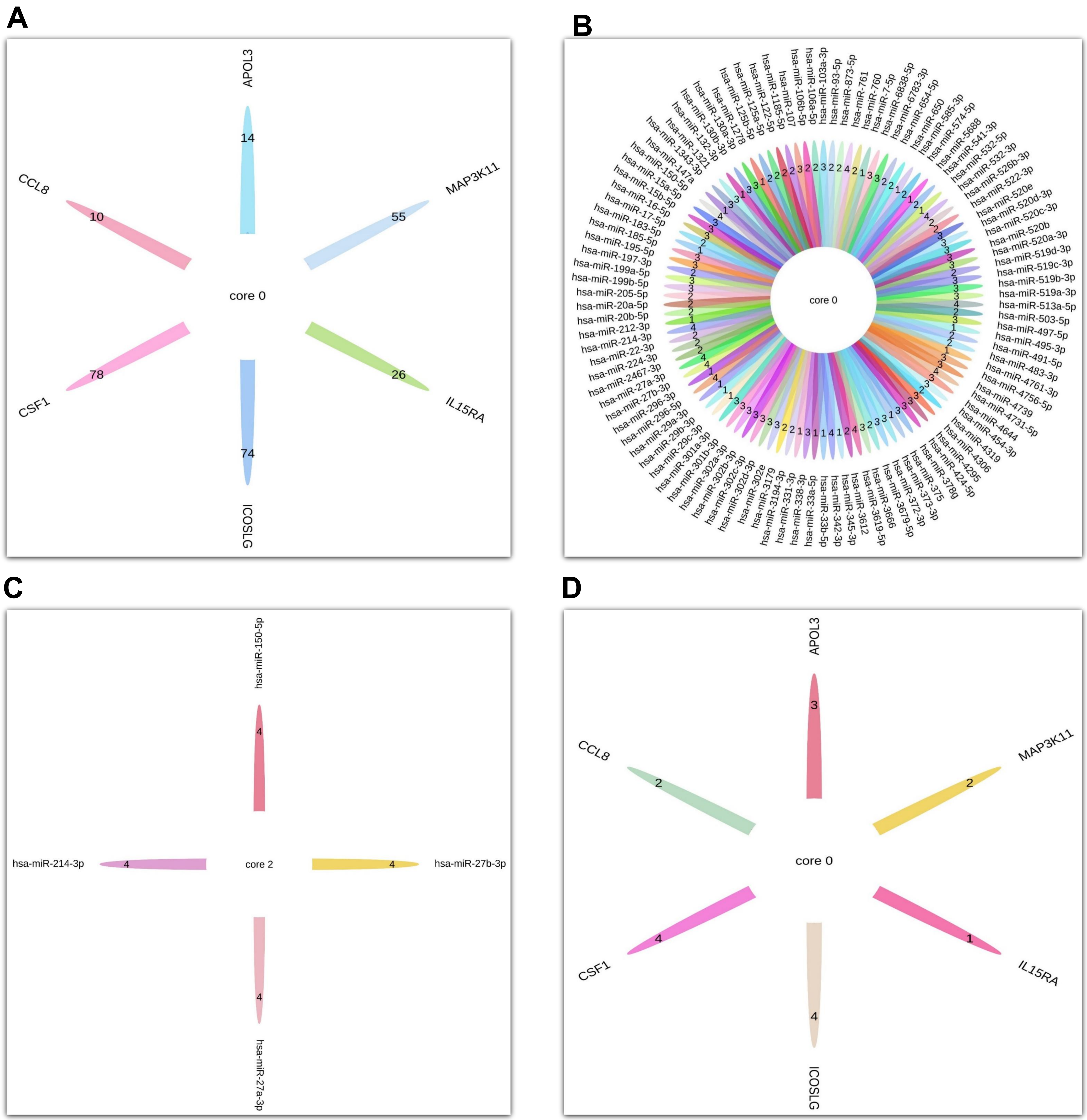
A
APOL3
14
MAP3K11
55
IL15RA
26
ICOSLG
74
CSF1
78
CCL8
10
core 0
B
core 0
hsa-miR-103a-3p
hsa-miR-93-5p
hsa-miR-873-5p
hsa-miR-761
hsa-miR-760
hsa-miR-7-5p
hsa-miR-6838-5p
hsa-miR-6783-3p
hsa-miR-654-5p
hsa-miR-650
hsa-miR-585-3p
hsa-miR-574-5p
hsa-miR-5688
hsa-miR-541-3p
hsa-miR-532-5p
hsa-miR-532-3p
hsa-miR-526b-3p
hsa-miR-522-3p
hsa-miR-520e
hsa-miR-520d-3p
hsa-miR-520c-3p
hsa-miR-520b
hsa-miR-520a-3p
hsa-miR-519d-3p
hsa-miR-519c-3p
hsa-miR-519b-3p
hsa-miR-519a-3p
hsa-miR-513a-5p
hsa-miR-503-5p
hsa-miR-497-5p
hsa-miR-495-3p
hsa-miR-491-5p
hsa-miR-483-3p
hsa-miR-4761-3p
hsa-miR-4756-5p
hsa-miR-4739
hsa-miR-4731-5p
hsa-miR-4644
hsa-miR-454-3p
hsa-miR-4319
hsa-miR-4306
hsa-miR-4295
hsa-miR-424-5p
hsa-miR-378g
hsa-miR-375
hsa-miR-373-3p
hsa-miR-372-3p
hsa-miR-3679-5p
hsa-miR-3666
hsa-miR-3619-5p
hsa-miR-3612
hsa-miR-345-3p
hsa-miR-342-3p
hsa-miR-33b-5p
hsa-miR-33a-5p
hsa-miR-338-3p
hsa-miR-331-3p
hsa-miR-3194-3p
hsa-miR-3179
hsa-miR-302e
hsa-miR-302d-3p
hsa-miR-302c-3p
hsa-miR-302b-3p
hsa-miR-302a-3p
hsa-miR-301b-3p
hsa-miR-301a-3p
hsa-miR-29c-3p
hsa-miR-29b-3p
hsa-miR-29a-3p
hsa-miR-296-5p
hsa-miR-296-3p
hsa-miR-27b-3p
hsa-miR-27a-3p
hsa-miR-2467-3p
hsa-miR-224-3p
hsa-miR-22-3p
hsa-miR-214-3p
hsa-miR-212-3p
hsa-miR-20b-5p
hsa-miR-20a-5p
hsa-miR-205-5p
hsa-miR-199b-5p
hsa-miR-199a-5p
hsa-miR-197-3p
hsa-miR-195-5p
hsa-miR-185-5p
hsa-miR-183-5p
hsa-miR-17-5p
hsa-miR-16-5p
hsa-miR-15b-5p
hsa-miR-15a-5p
hsa-miR-150-5p
hsa-miR-147a
hsa-miR-1343-3p
hsa-miR-1321
hsa-miR-132-3p
hsa-miR-130b-3p
hsa-miR-130a-3p
hsa-miR-1278
hsa-miR-125b-5p
hsa-miR-125a-5p
hsa-miR-122-5p
hsa-miR-1185-5p
hsa-miR-107
hsa-miR-106b-5p
hsa-miR-106a-5p
C
hsa-miR-150-5p
4
hsa-miR-27b-3p
4
hsa-miR-27a-3p
4
hsa-miR-214-3p
4
core 2
D
APOL3
3
MAP3K11
2
IL15RA
1
ICOSLG
4
CSF1
4
CCL8
2
core 0

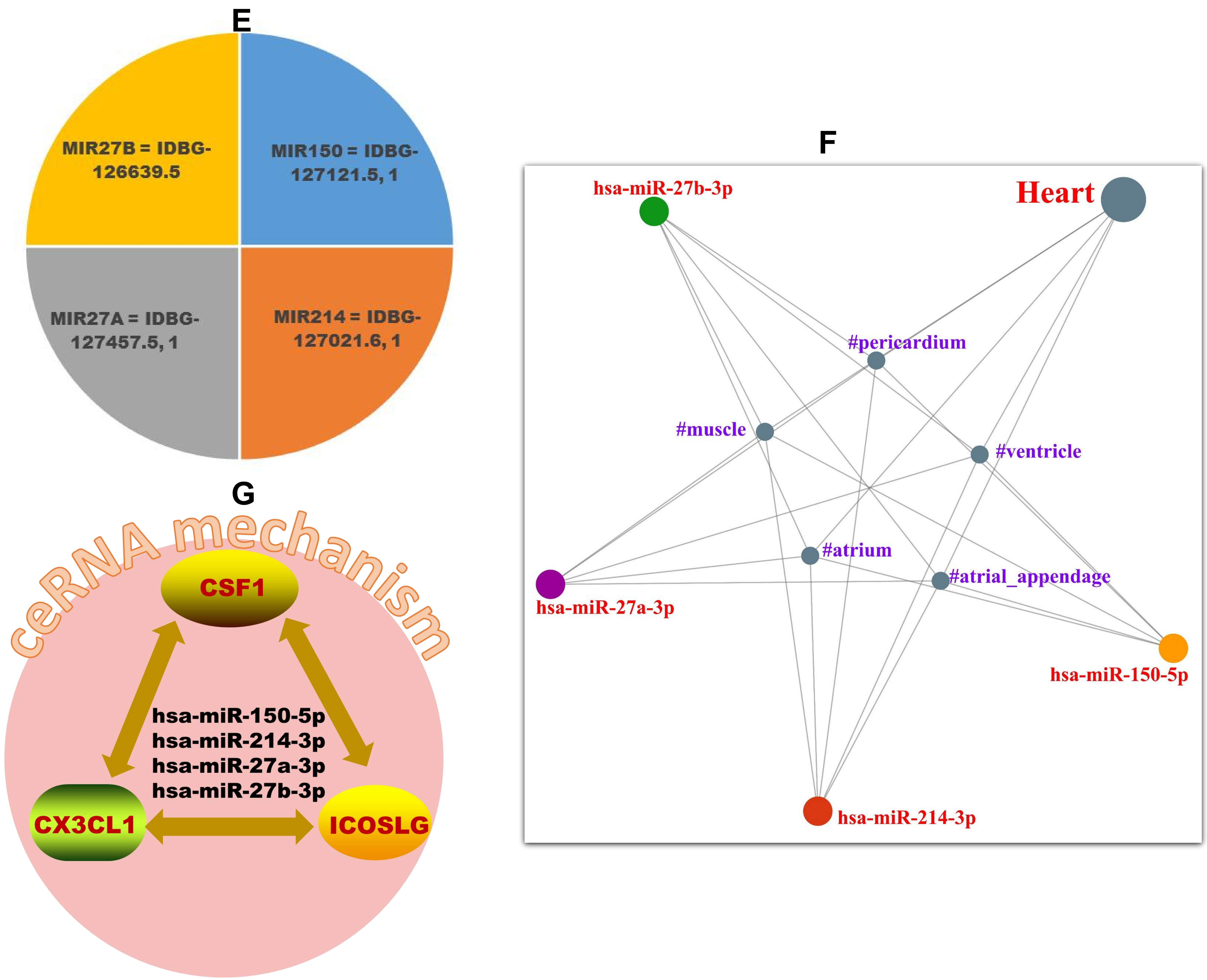
E
MIR27B = IDBG-126639.5
MIR150 = IDBG-127121.5, 1
MIR27A = IDBG-127457.5, 1
MIR214 = IDBG-127021.6, 1
F
hsa-miR-27b-3p
Heart
#pericardium
#muscle
#ventricle
#atrium
#atrial_appendage
hsa-miR-27a-3p
hsa-miR-150-5p
hsa-miR-214-3p
G
ceRNA mechanism
CSF1
hsa-miR-150-5p
hsa-miR-214-3p
hsa-miR-27a-3p
hsa-miR-27b-3p
CX3CL1
ICOSLG

Supplementary Figure S4. Key miRNAs underlying ceRNA mechanism of CX3CL1. (A) Number of miRNAs of six intersection genes form ceRNA with CX3CL1. (B,C) miRNAs shared by intersection genes. (D) Number of intersection genes shared by the four miRNAs. (E) The indicated miRNAs were annotated in InnateDB. (F) Regulatory network of the four miRNA in heart. (G) Elements of ceRNA network obtained by all sorts of filtrations.

FIG.S5

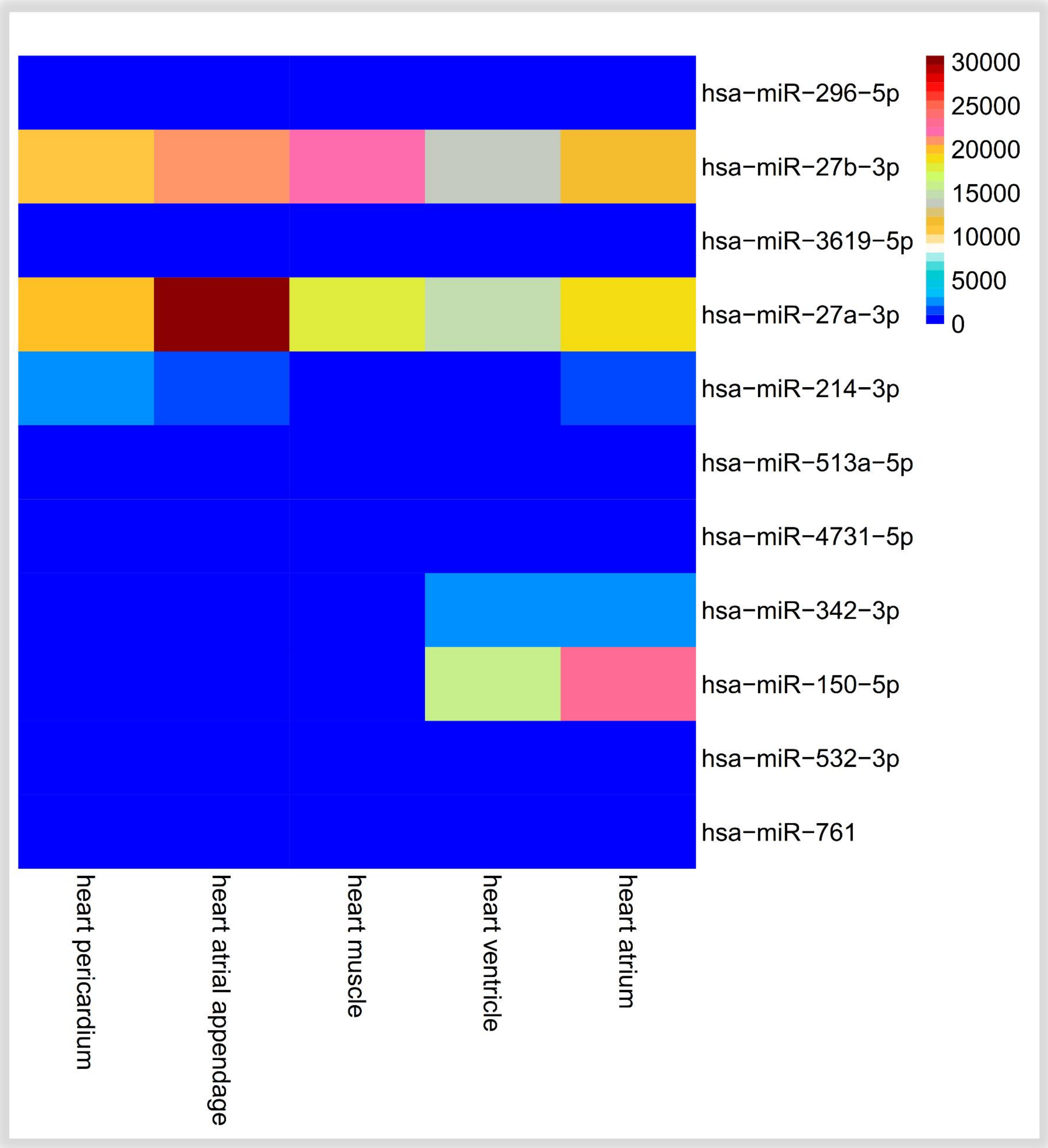

hsa−miR−296−5p
hsa−miR−27b−3p
hsa−miR−3619−5p
hsa−miR−27a−3p
hsa−miR−214−3p
hsa−miR−513a−5p
hsa−miR−4731−5p
hsa−miR−342−3p
hsa−miR−150−5p
hsa−miR−532−3p
hsa−miR−761
30000
25000
20000
15000
10000
5000
0
heart pericardium
heart atrial appendage
heart muscle
heart ventricle
heart atrium

Supplementary Figure S5. Expression profiling of the intersection miRNAs in all parts of heart Expressions of hsa-miR-150-5p, hsa-miR-214-3p,hsa-miR-27a-3p,hsa-miR-27b-3p,hsa-miR-296-5p, hsa-miR-342-3p, hsa-miR-3619-5p, hsa-miR-4731-5p, hsa-miR-513a-5p,hsa-miR-532-3p,hsa-miR-761 .

FIG.S6

mirPath v.3

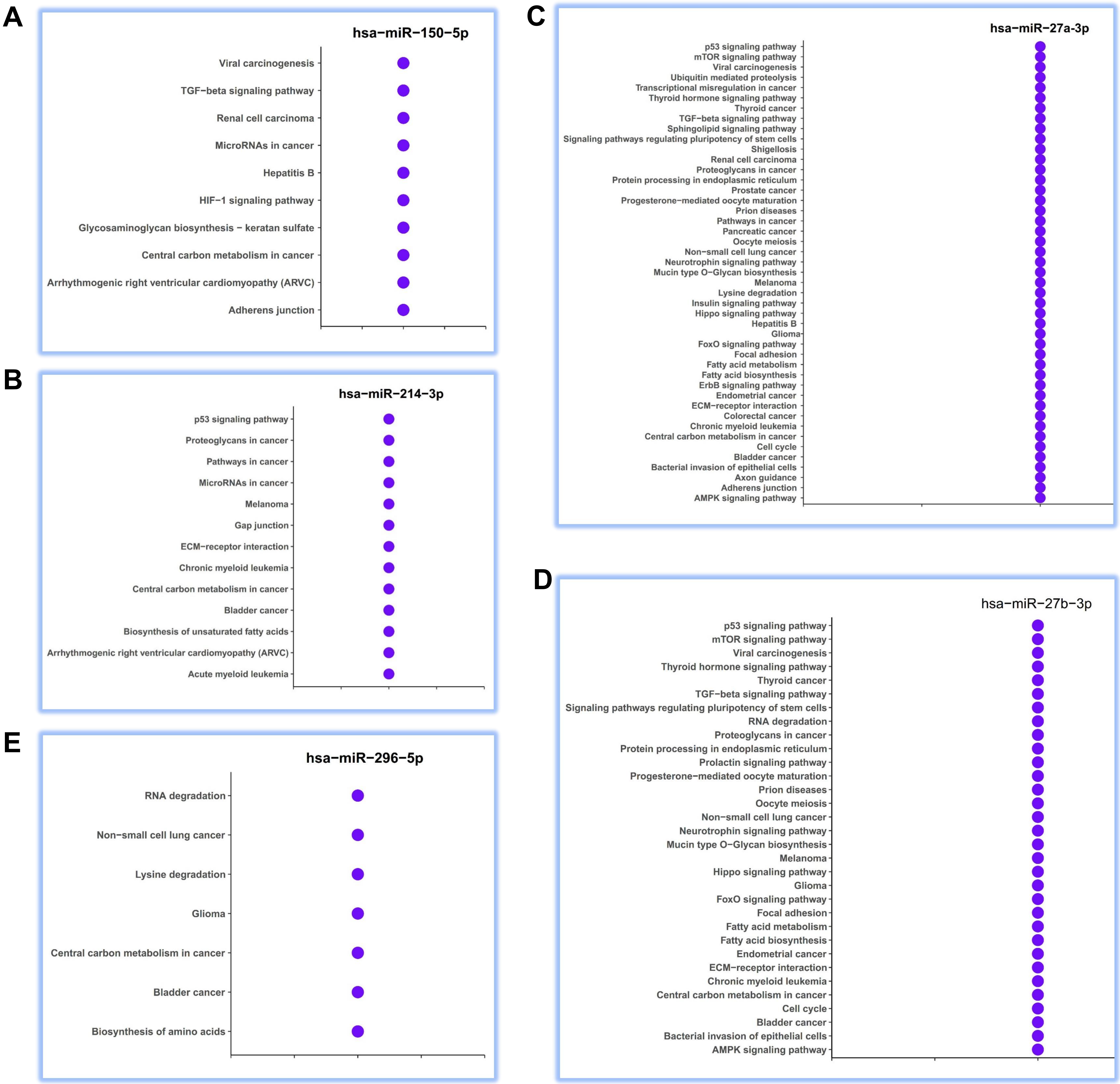

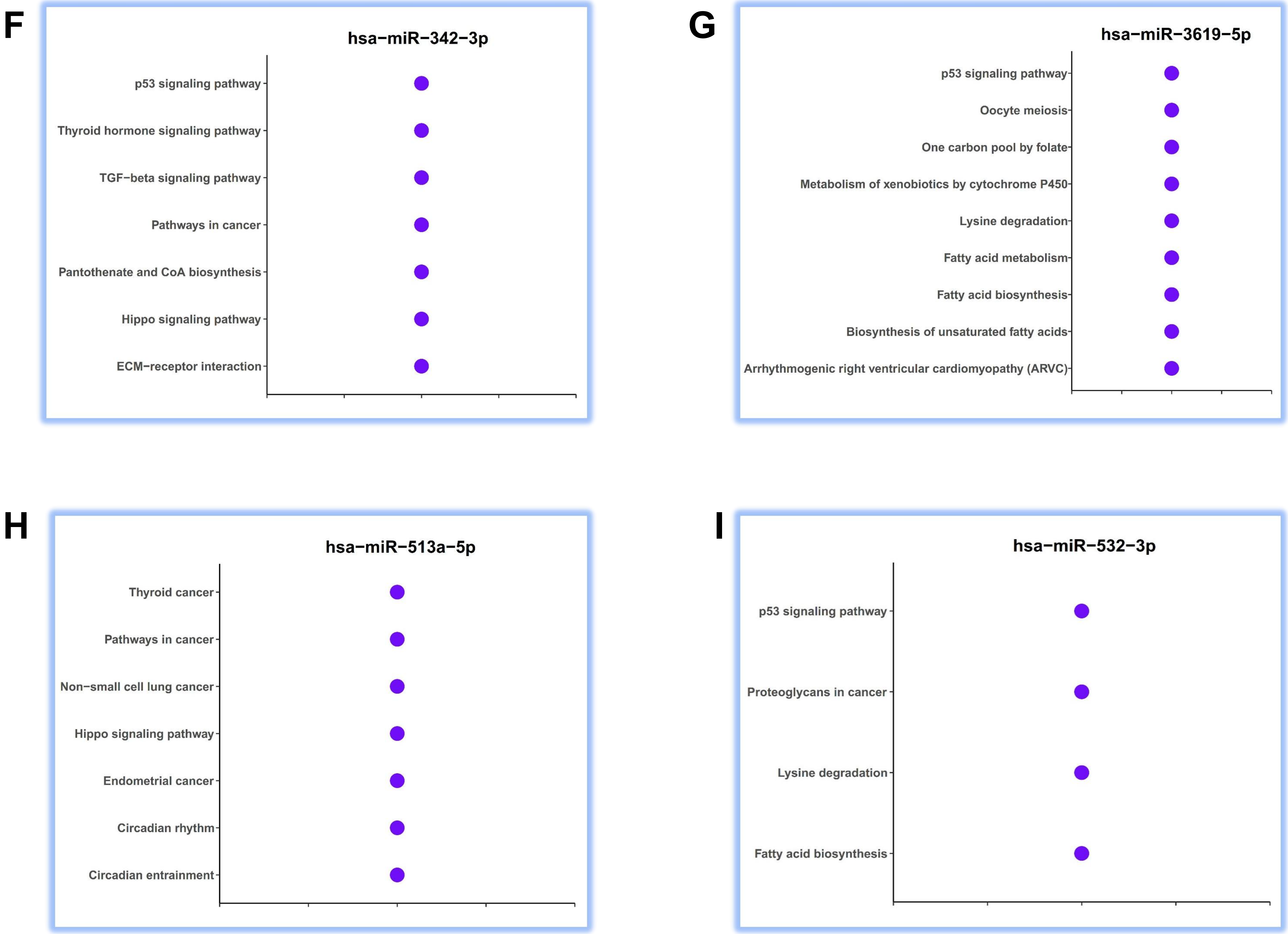
F
hsa−miR−342−3p
p53 signaling pathway
Thyroid hormone signaling pathway
TGF−beta signaling pathway
Pathways in cancer
Pantothenate and CoA biosynthesis
Hippo signaling pathway
ECM−receptor interaction
G
hsa−miR−3619−5p
p53 signaling pathway
Oocyte meiosis
One carbon pool by folate
Metabolism of xenobiotics by cytochrome P450
Lysine degradation
Fatty acid metabolism
Fatty acid biosynthesis
Biosynthesis of unsaturated fatty acids
Arrhythmogenic right ventricular cardiomyopathy (ARVC)
H
hsa−miR−513a−5p
Thyroid cancer
Pathways in cancer
Non−small cell lung cancer
Hippo signaling pathway
Endometrial cancer
Circadian rhythm
Circadian entrainment
I
hsa−miR−532−3p
p53 signaling pathway
Proteoglycans in cancer
Lysine degradation
Fatty acid biosynthesis

Supplementary Figure S6. KEGG pathway annotations of the intersection miRNAs (A) Functional notes of hsa-miR-150-5p. (B) Functional notes of hsa-miR-214-3p. (C) Functional notes of hsa-miR-27a-3p. (D) Functional notes of hsa-miR-27b-3p. (E) Functional notes of hsa-miR-296-5p. (F) Functional notes of hsa-miR-342-3p. (G) Functional notes of hsa-miR-3619-5p. (H) Functional notes of hsa-miR-513a-5p. (I) Functional notes of hsa-miR-532-3p.

FIG.S7

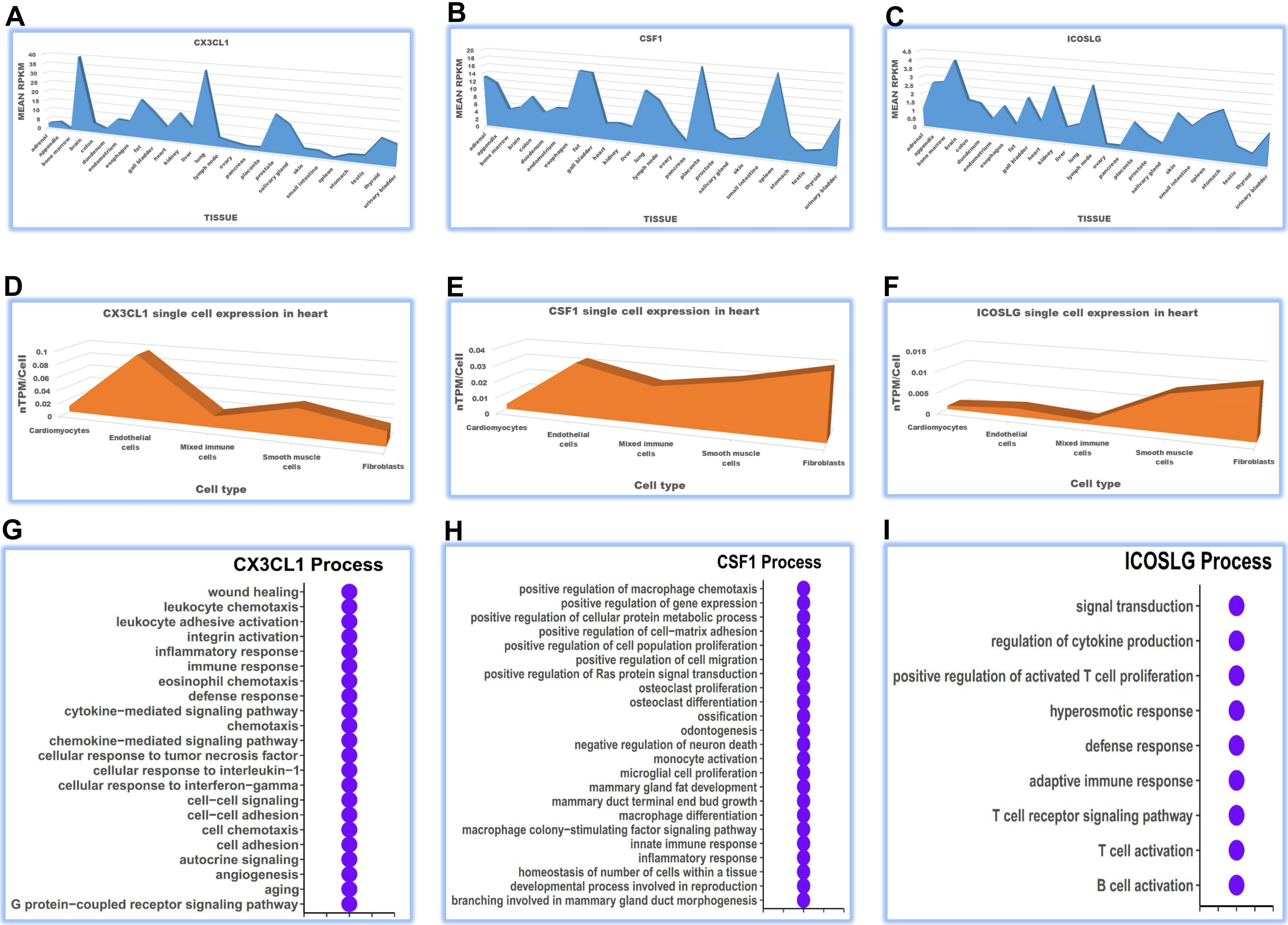
A
CX3CL1
MEAN RPKM
TISSUE
B
CSF1
MEAN RPKM
TISSUE
C
ICOSLG
MEAN RPKM
TISSUE
adrenal
appendix
bone marrow
brain
colon
duodenum
endometrium
esophagus
fat
gall bladder
heart
kidney
liver
lung
lymph node
ovary
pancreas
placenta
prostate
salivary gland
skin
small intestine
spleen
stomach
testis
thyroid
urinary bladder
D
CX3CL1 single cell expression in heart
E
CSF1 single cell expression in heart
F
ICOSLG single cell expression in heart
nTPM/Cell
Cardiomyocytes
Endothelial cells
Mixed immune cells
Smooth muscle cells
Fibroblasts
Cell type
G
CX3CL1 Process
wound healing
leukocyte chemotaxis
leukocyte adhesive activation
integrin activation
inflammatory response
immune response
eosinophil chemotaxis
defense response
cytokine−mediated signaling pathway
chemotaxis
chemokine−mediated signaling pathway
cellular response to tumor necrosis factor
cellular response to interleukin−1
cellular response to interferon−gamma
cell−cell signaling
cell−cell adhesion
cell chemotaxis
cell adhesion
autocrine signaling
angiogenesis
aging
G protein−coupled receptor signaling pathway
H
CSF1 Process
positive regulation of macrophage chemotaxis
positive regulation of gene expression
positive regulation of cellular protein metabolic process
positive regulation of cell−matrix adhesion
positive regulation of cell population proliferation
positive regulation of cell migration
positive regulation of Ras protein signal transduction
osteoclast proliferation
osteoclast differentiation
ossification
odontogenesis
negative regulation of neuron death
monocyte activation
microglial cell proliferation
mammary gland fat development
mammary duct terminal end bud growth
macrophage differentiation
macrophage colony−stimulating factor signaling pathway
innate immune response
inflammatory response
homeostasis of number of cells within a tissue
developmental process involved in reproduction
branching involved in mammary gland duct morphogenesis
I
ICOSLG Process
signal transduction
regulation of cytokine production
positive regulation of activated T cell proliferation
hyperosmotic response
defense response
adaptive immune response
T cell receptor signaling pathway
T cell activation
B cell activation

Supplementary Figure S7. Prospect annotations of the key coding genes (A-C) Tissue expression profiling of CX3CL1, CSF1, and ICOSLG, respectively. (D-F) Single cell expression profiling of CX3CL1, CSF1, and ICOSLG, respectively. (G-I) Functional process of CX3CL1, CSF1, and ICOSLG.

FIG.S8

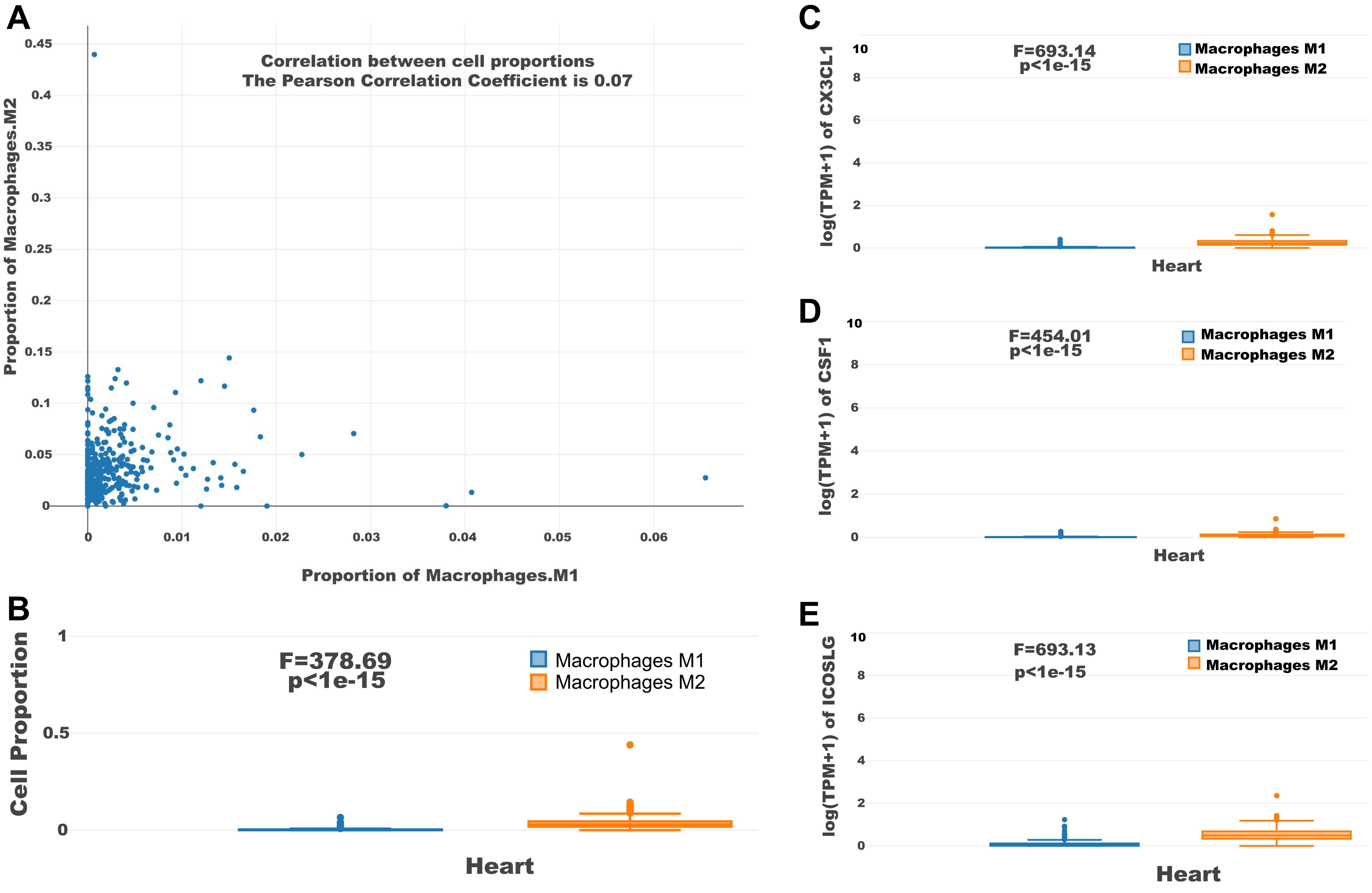
A
Correlation between cell proportions
The Pearson Correlation Coefficient is 0.07
Proportion of Macrophages.M2
Proportion of Macrophages.M1
0.45
0.4
0.35
0.3
0.25
0.2
0.15
0.1
0.05
0
0.01
0.02
0.03
0.04
0.05
0.06
B
Cell Proportion
F=378.69
p<1e-15
Macrophages M1
Macrophages M2
1
0.5
Heart
C
log(TPM+1) of CX3CL1
F=693.14
p<1e-15
Macrophages M1
Macrophages M2
10
8
6
4
2
Heart
D
log(TPM+1) of CSF1
F=454.01
p<1e-15
Macrophages M1
Macrophages M2
Heart
E
log(TPM+1) of ICOSLG
F=693.13
p<1e-15
Macrophages M1
Macrophages M2
Heart

Supplementary Figure S8. Correlations between CX3CL1-centered network molecules and macrophages in heart (A) Correlation between M1 and M2 proportion. (B) Differential analysis for M1 and M2 proportion. (C) Differential expression analysis for CX3CL1 in M1 and M2 cells. (D) Differential expression analysis for CSF1 in M1 and M2 cells. (E) Differential expression analysis for ICOSLG in M1 and M2 cells.

FIG.S9

A

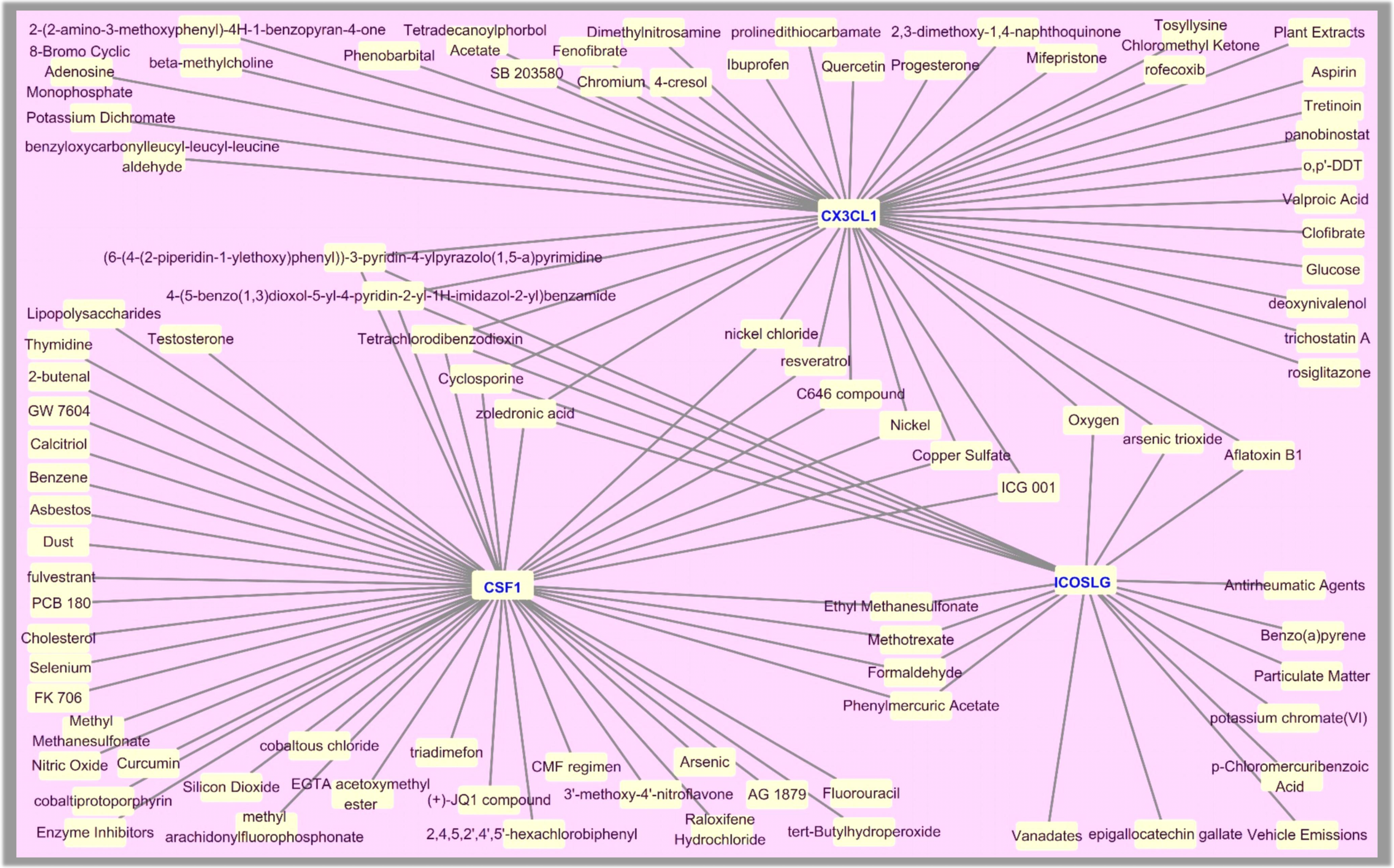

CX3CL1
CSF1
ICOSLG
2-(2-amino-3-methoxyphenyl)-4H-1-benzopyran-4-one
8-Bromo Cyclic Adenosine Monophosphate
beta-methylcholine
Potassium Dichromate
benzyloxycarbonylleucyl-leucyl-leucine aldehyde
Tetradecanoylphorbol Acetate
Phenobarbital
SB 203580
Fenofibrate
Chromium
4-cresol
Dimethylnitrosamine
prolinedithiocarbamate
Ibuprofen
Quercetin
2,3-dimethoxy-1,4-naphthoquinone
Progesterone
Mifepristone
Tosyllysine Chloromethyl Ketone
rofecoxib
Plant Extracts
Aspirin
Tretinoin
panobinostat
o,p'-DDT
Valproic Acid
Clofibrate
Glucose
deoxynivalenol
trichostatin A
rosiglitazone
(6-(4-(2-piperidin-1-ylethoxy)phenyl))-3-pyridin-4-ylpyrazolo(1,5-a)pyrimidine
4-(5-benzo(1,3)dioxol-5-yl-4-pyridin-2-yl-1H-imidazol-2-yl)benzamide
Lipopolysaccharides
Testosterone
Tetrachlorodibenzodioxin
nickel chloride
resveratrol
C646 compound
Cyclosporine
zoledronic acid
Nickel
Copper Sulfate
ICG 001
Oxygen
arsenic trioxide
Aflatoxin B1
Thymidine
2-butenal
GW 7604
Calcitriol
Benzene
Asbestos
Dust
fulvestrant
PCB 180
Cholesterol
Selenium
FK 706
Methyl Methanesulfonate
Nitric Oxide
Curcumin
cobaltous chloride
triadimefon
cobaltiprotoporphyrin
Silicon Dioxide
EGTA acetoxymethyl ester
(+)-JQ1 compound
CMF regimen
Arsenic
3'-methoxy-4'-nitroflavone
AG 1879
Fluorouracil
Enzyme Inhibitors
methyl arachidonylfluorophosphonate
2,4,5,2',4',5'-hexachlorobiphenyl
Raloxifene Hydrochloride
tert-Butylhydroperoxide
Ethyl Methanesulfonate
Methotrexate
Formaldehyde
Phenylmercuric Acetate
Antirheumatic Agents
Benzo(a)pyrene
Particulate Matter
potassium chromate(VI)
p-Chloromercuribenzoic Acid
Vanadates
epigallocatechin gallate
Vehicle Emissions

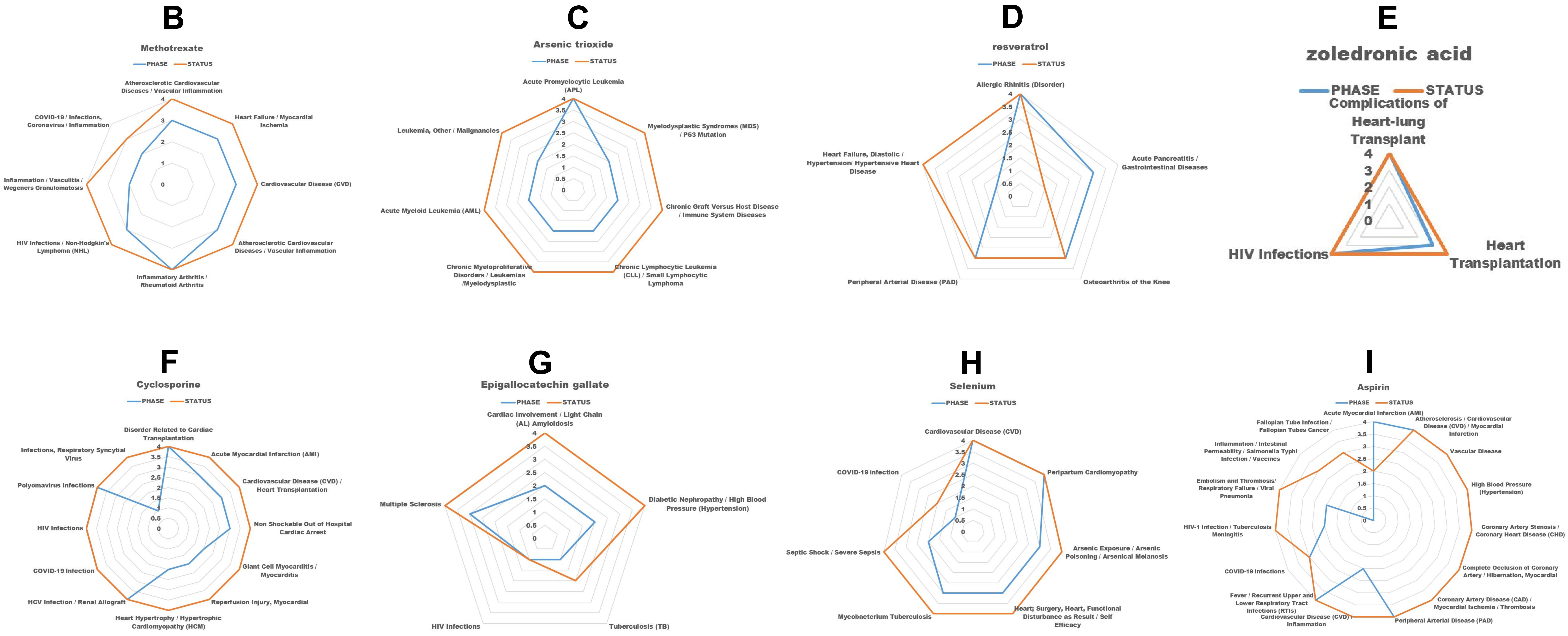

B
Methotrexate
PHASE STATUS
Atherosclerotic Cardiovascular Diseases / Vascular Inflammation
Heart Failure / Myocardial Ischemia
Cardiovascular Disease (CVD)
Atherosclerotic Cardiovascular Diseases / Vascular Inflammation
Inflammatory Arthritis / Rheumatoid Arthritis
HIV Infections / Non-Hodgkin's Lymphoma (NHL)
Inflammation / Vasculitis / Wegeners Granulomatosis
COVID-19 / Infections, Coronavirus / Inflammation
C
Arsenic trioxide
PHASE STATUS
Acute Promyelocytic Leukemia (APL)
Myelodysplastic Syndromes (MDS) / P53 Mutation
Chronic Graft Versus Host Disease / Immune System Diseases
Chronic Lymphocytic Leukemia (CLL) / Small Lymphocytic Lymphoma
Chronic Myeloproliferative Disorders / Leukemias /Myelodysplastic
Acute Myeloid Leukemia (AML)
Leukemia, Other / Malignancies
D
resveratrol
PHASE STATUS
Allergic Rhinitis (Disorder)
Acute Pancreatitis / Gastrointestinal Diseases
Osteoarthritis of the Knee
Peripheral Arterial Disease (PAD)
Heart Failure, Diastolic / Hypertension/ Hypertensive Heart Disease
E
zoledronic acid
PHASE STATUS
Complications of Heart-lung Transplant
Heart Transplantation
HIV Infections
F
Cyclosporine
PHASE STATUS
Disorder Related to Cardiac Transplantation
Acute Myocardial Infarction (AMI)
Cardiovascular Disease (CVD) / Heart Transplantation
Non Shockable Out of Hospital Cardiac Arrest
Giant Cell Myocarditis / Myocarditis
Reperfusion Injury, Myocardial
Heart Hypertrophy / Hypertrophic Cardiomyopathy (HCM)
HCV Infection / Renal Allograft
COVID-19 Infection
HIV Infections
Polyomavirus Infections
Infections, Respiratory Syncytial Virus
G
Epigallocatechin gallate
PHASE STATUS
Cardiac Involvement / Light Chain (AL) Amyloidosis
Diabetic Nephropathy / High Blood Pressure (Hypertension)
Tuberculosis (TB)
HIV Infections
Multiple Sclerosis
H
Selenium
PHASE STATUS
Cardiovascular Disease (CVD)
Peripartum Cardiomyopathy
Arsenic Exposure / Arsenic Poisoning / Arsenical Melanosis
Heart; Surgery, Heart, Functional Disturbance as Result / Self Efficacy
Mycobacterium Tuberculosis
Septic Shock / Severe Sepsis
COVID-19 infection
I
Aspirin
PHASE STATUS
Acute Myocardial Infarction (AMI)
Atherosclerosis / Cardiovascular Disease (CVD) / Myocardial Infarction
Vascular Disease
High Blood Pressure (Hypertension)
Coronary Artery Stenosis / Coronary Heart Disease (CHD)
Complete Occlusion of Coronary Artery / Hibernation, Myocardial
Coronary Artery Disease (CAD) / Myocardial Ischemia / Thrombosis
Peripheral Arterial Disease (PAD)
Cardiovascular Disease (CVD) / Inflammation
Fever / Recurrent Upper and Lower Respiratory Tract Infections (RTIs)
COVID-19 Infections
HIV-1 Infection / Tuberculosis Meningitis
Embolism and Thrombosis/ Respiratory Failure / Viral Pneumonia
Inflammation / Intestinal Permeability / Salmonella Typhi Infection / Vaccines
Fallopian Tube Infection / Fallopian Tubes Cancer

Supplementary Figure S9. Chemicals (drugs) act on crucial coding genes (A) Ninety chemicals targeted the three coding genes. (B) Clinical trials of Methotrexate. (C) Clinical trials of Arsenic trioxide. (D) Clinical trials of Resveratrol. (E) Clinical trials of Zoledronic acid. (F) Clinical trials of Cyclosporine. (G) Clinical trials of Epigallocatechin gallate. (H) Clinical trials of Selenium. (I) Clinical trials of Aspirin.
